# Blocking Oncostatin M receptor abrogates STAT3 mediated integrin signaling and overcomes chemoresistance in ovarian cancer

**DOI:** 10.1038/s41698-024-00593-y

**Published:** 2024-06-05

**Authors:** Anjali Geethadevi, Zhiqiang Ku, Shirng-Wern Tsaih, Deepak Parashar, Ishaque P. Kadamberi, Wei Xiong, Hui Deng, Jasmine George, Sudhir Kumar, Sonam Mittal, Ningyan Zhang, Sunila Pradeep, Zhiqiang An, Pradeep Chaluvally-Raghavan

**Affiliations:** 1 Department of Obstetrics and Gynecology, Medical College of Wisconsin, Milwaukee, WI USA; 2 Medical College of Wisconsin Cancer Center, Medical College of Wisconsin, Milwaukee, WI USA; 3 Texas Therapeutics Institute, Brown Foundation Institute of Molecular Medicine, The University of Texas Health Science Center, Houston, TX USA; 4 Department of Medicine, Division of Hematology & Oncology, Medical College of Wisconsin, Milwaukee, WI USA; 5 Department of Physiology, Medical College of Wisconsin, Milwaukee, WI USA

**Keywords:** Ovarian cancer, Cancer

## Abstract

Chemotherapy such as cisplatin is widely used to treat ovarian cancer either before or after surgical debulking. However, cancer relapse due to chemotherapy resistance is a major challenge in the treatment of ovarian cancer. The underlying mechanisms related to chemotherapy resistance remain largely unclear. Therefore, identification of effective therapeutic strategies is urgently needed to overcome therapy resistance. Transcriptome-based analysis, in vitro studies and functional assays identified that cisplatin-resistant ovarian cancer cells express high levels of OSMR compared to cisplatin sensitive cells. Furthermore, OSMR expression associated with a module of integrin family genes and predominantly linked with integrin αV (ITGAV) and integrin β3 (ITGB3) for cisplatin resistance. Using ectopic expression and knockdown approaches, we proved that OSMR directly regulates ITGAV and ITGB3 gene expression through STAT3 activation. Notably, targeting OSMR using anti-OSMR human antibody inhibited the growth and metastasis of ovarian cancer cells and sensitized cisplatin treatment. Taken together, our results underscore the pivotal role of OSMR as a requirement for cisplatin resistance in ovarian cancer. Notably, OSMR fostered the expression of a distinct set of integrin genes, which in turn resulted into a crosstalk between OSMR and integrins for signaling activation that is critical for cisplatin resistance. Therefore, targeting OSMR emerges as a promising and viable strategy to reverse cisplatin-resistance in ovarian cancer.

## Introduction

Ovarian cancer is the most fatal gynecological malignancy, where 75–80% of the patients display advanced disease with a relapse within three years mostly due to therapy resistance. Most women are diagnosed with ovarian cancer in later stages, with a five-year survival rate of only about 39% due to chemotherapy resistance and subsequent recurrence^1^. As chemoresistance remains a major challenge in the treatment of ovarian cancer, understanding the molecular mechanisms responsible for chemoresistance is critical for developing effective therapeutic strategies to combine with the current therapy and to achieve synergistic effects in the clinical settings.

Studies have reported the role of interleukin-6 (IL6)-family of cytokines and its downstream oncogenic mechanisms such as JAK/STAT3, PI3K/AKT, and ERK signaling in chemoresistant mechanisms^2,3^. However, Oncostatin M (OSM) and its receptor Oncostatin M receptor (OSMR) are poorly studied for chemoresistance mechanisms. OSM is one of the IL6-family proteins, which binds to its receptor OSMR, and heterodimerizes with GP130 (a.k.a. IL6ST) or Interleukin-31 receptor (IL31RA) then activates downstream signaling cues such as JAK1/JAK2-STAT, PI3K/AKT and MEK/ERK pathways^4^. Several studies including ours have demonstrated the pro-tumorigenic role of OSM and its receptor OSMR in solid tumors such as breast cancer^5,6^, pancreatic cancer^7,8^, endometrial cancer^9^, cervical cancer^10^ and ovarian cancer^11^ and their contribution to cancer aggressiveness and metastasis, cancer stemness, epithelial to mesenchymal transition (EMT)^8,12^, inflammatory response^13^, and angiogenesis^14^. It is also reported that high OSMR expression is associated with poor prognosis in several cancers and in inflammatory bowel disease patients^7,10,15,16^. Our recent work has reported that OSMR is a critical regulator of oncogenic addiction mechanism in ovarian cancer cells. We have further shown that OSMR activation is mainly orchestrated through both autocrine and paracrine production of OSM, where OSM is predominantly produced by tumor-associated macrophages (TAM)^11^.

Tumor cell adhesion and proliferation at the newly seeded sites depends on a large family of cellular adhesion receptors known as integrins (ITG). Integrins are activated upon interaction of integrin α and β with the respective ligands such as laminin, fibronectin, collagen, vitronectin, etc. which are part of extracellular matrix (ECM)^17–19^. Mainly, 24 different subtypes of integrins, composed of α and β subunits, have been identified so far^17–19^. Our studies have identified multiple integrins such as ITGAV, ITGA3, ITGB1, ITGB3, ITGB6, and ITGB8 which are upregulated in association with OSM signaling activated through OSMR. On this background, we have characterized how the integrin signaling is regulated by OSMR in ovarian cancer cells and the potential of inhibiting OSMR for cisplatin sensitization. We have developed a novel antibody that binds to OSMR and blocks the signaling activated through OSM by promoting internalization and degradation of OSMR^11^. We have also reported that targeting OSMR is an efficient approach to treat ovarian cancer^11^. In the current study, we determined the potential of using our anti-OSMR antibody to sensitize cisplatin treatment to treat cisplatin-resistant ovarian cancer.

## Results

### Oncostatin M and its receptor OSMR are upregulated in cisplatin-resistant ovarian cancer cells

IL-6 family of inflammatory cytokines are known to be associated with the cancer progression and chemoresistance in many malignancies including epithelial ovarian cancer^20,21^. Our recent research reported that OSM-mediated signaling activation through OSMR dimerization with IL6ST is important for Signaling Transducer and Activator of Transcription 3 (STAT3), which is known for oncogenic transformation and blocks apoptosis in both breast and ovarian cancers^5,11^. Therefore, we sought to determine if OSMR is highly expressed in cisplatin-resistant cells and identify the genes and pathways which are associated with OSMR. Towards this aim, we employed two publicly available microarray gene expression datasets (GSE45553 and GSE33482) consisting of gene expression data of cisplatin sensitive and resistant OVCAR8 spheroids, and cisplatin sensitive and resistant A2780 cells respectively^22^.

**Fig. 1:**
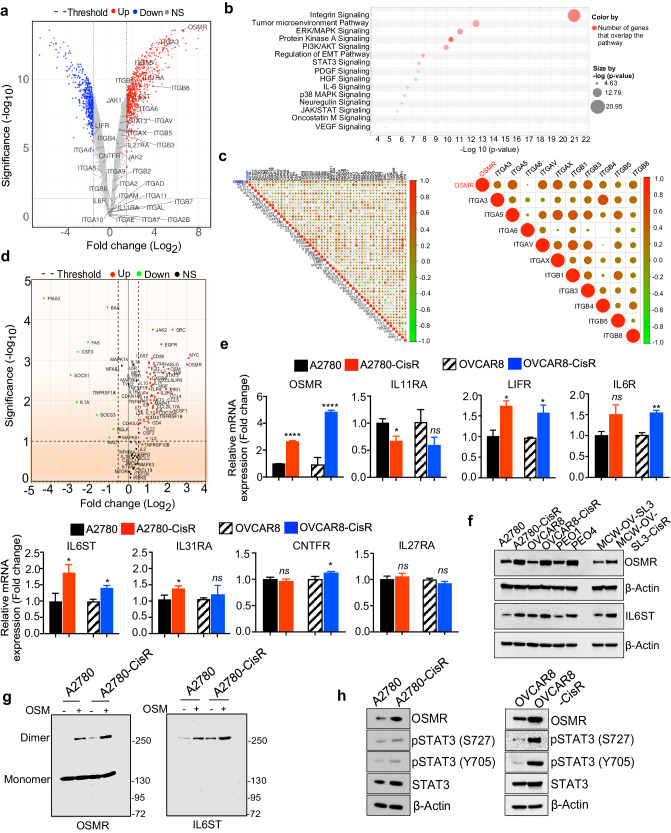
Oncostatin M (OSM) receptor is upregulated in chemoresistant ovarian cancer through OSMR heterodimerization. **a** Volcano plot shows differentially expressed genes in OVCAR8-CisR (cisplatin resistant) spheroids (*N* = 4) compared with OVCAR8 sensitive spheroids (*N* = 4) in GSE45553 microarray dataset. Significance threshold is *P* ≤ 0.05 and Log2 (fold change) threshold set at ±0.585 on both sides. Top hits and OSM module genes are labeled (*P* value < 0.05, FDR < 0.1). ns non-significant. **b** Ingenuity Pathway Analysis (IPA) shows canonical pathways that are >1.5-fold change in OVCAR8-CisR spheroids (GSE45553) with a threshold cut off log10 (fold change) > 4 respectively. **c** Correlation graph shows Spearman’s correlation of OSMR with (left panel) Integrin family genes and (right panel) with integrins α and β genes exhibiting *P* value ≤ 0.05 in TCGA ovarian cancer cohort (*n* = 379) dataset downloaded from GDC data portal. **d** Volcano plot shows differentially expressed genes in A2780 CisR cell line compared with A2780 sensitive cell lines in triplicates in the qPCR array panel of IL6 signaling. Genes were selected based on *P* value ≤ 0.05 and Log2 (fold change) threshold set at +/−0.58 in both directions. ns: non-significant. **e** Total RNA was isolated from the indicated c**e**ll lines and mRNA expression of IL6 receptor family genes were determined. β-actin gene was used as an internal standard. **f** Western blot shows OSMR and IL6ST expression in the lysates of a set of cisplatin-sensitive and resistant ovarian cancer cell lines. **g** OSMR was immunoprecipitated (IP) from A2780-CisR and A2780 sensitive cells were treated with recombinant human OSM (100 ng/mL) and crosslinked with BS3 agent. Monomers and dimers obtained by IP were resolved on SDS-PAGE and immunoblotted using OSMR and IL6ST specific antibodies. **h** Western blot shows the level of OSMR, and total and phosphorylated form of STAT3 in both cisplatin sensitive and resistant version of A2780 and OVCAR8 cells. Student’s *t* test (two tailed, unpaired) was performed to determine significance between the groups. Error bars represent mean ± SEM. ****P ≤ 0.0001, **P ≤ 0.01, *P ≤ 0.05, ns non-significant.

Notably, OSMR and IL-6 genes were identified within the top-15 genes which are highly expressed in the dataset of cisplatin-resistant OVCAR8 (OVCAR8-CisR) spheroids (GSE45553) (Fig. 1a). Further, we identified that integrin signaling, tumor microenvironment pathways, ERK/MAPK pathways, regulation of EMT, STAT3 signaling etc. were the highly enriched pathways in the differentially upregulated genes in the OVCAR8-CisR spheroid dataset (Fig. 1b). Notably, integrin signaling was the most outperforming pathway with a lowest *P* value among the identified pathways (Fig. 1b). However, the association of OSMR-signaling with integrins and the crosstalk between OSMR and integrin signaling were not well established before in cancer and particularly in the context of cisplatin resistance. Therefore, we analyzed the correlation of OSMR with integrin family genes (obtained from IPA analysis) (left panel) and integrins α and β genes (right panel) in the TCGA-OV ovarian cancer cohort (*n* = 379) downloaded from TCGA-GDC portal (Fig. 1c) and in GSE45553 OVCAR8-CisR resistant spheroid datasets (Supplementary Fig. 1e, f; *P* ≤ 0.05). Importantly, OSMR exhibited high level of positive correlation with ITGAV, ITGA3, ITGA5, ITGB1, ITGB3, ITGB4, ITGB5 and ITGB8 with *P* value ≤0.05 in TCGA ovarian cancer cells (Fig. 1c). In GSE45553 OVCAR8-CisR spheroid datasets, OSMR was positively correlated with ITGA3, ITGA5, ITGB1, ITGB3, ITGB4, ITGB5, ITGB6 and ITGB8 with Spearman correlation *r* value ≥ 0.6 and a *P* value ≤ 0.05 and with ITGAV the Spearman correlation *r* value = 0.69 and a *P* value = 0.06 (Supplementary Fig. 1e, f).

In addition to our analysis displayed in Fig. 1a of microarray dataset of OVCAR8-CisR resistant spheroids (GSE45553), we used an additional dataset A2780-CisR resistant cell lines (GSE33482) with their respective parental version and found that many of the IL-6 family genes such as OSMR, interleukin-6 receptor (IL6R), leukemia inhibitory factor receptor (LIFR) are upregulated in the A2780-CisR resistant cell line (Supplementary Fig. 1a). We have identified 434 genes are commonly upregulated and 242 genes are commonly downregulated in cisplatin-resistant cell lines (P ≤0.05, FDR < 0.1; Supplementary Fig. 1c) with EMT, JAK/STAT, PI3K/AKT pathways as top commonly enriched pathways (Supplementary Fig. 1d) when both datasets were combined. We also validated this finding by comparing the expression of OSMR and other OSM family module genes in A2780-CisR resistant with cisplatin sensitive (parental) A2780 ovarian cancer cells by isolating RNA and performing qPCR array comprised of cytokines, interleukins, chemokines, their receptors and downstream effector genes. We found that 66 genes were differentially expressed in the A2780-CisR resistant ovarian cancer cells, where OSMR (Fold change: 8.28; *p* = 0.0013) was identified as one of the top genes among the genes were upregulated in A2780-CisR resistant cells which was consistent with our observation in the microarray datasets (Fig. 1d).

Among the 66 differentially expressed genes, we found c-MYC, OSMR, CSF1, SRC1, TNF-α, OSM, EGFR, FASLG, PIM1, and STAT3 were the top-10 genes upregulated in the A2780-CisR resistant cells. In contrast, the genes which are required for signaling attenuation such as SOCS1, and PIAS3 and pro-apoptotic genes BAX were downregulated in the A2780-CisR resistant cells (Fig. 1d). Notably, OSMR and its ligand OSM, and the downstream effectors such as JAK2 and STAT3 were expressed in A2780-CisR resistant cells compared to the control (Fig. 1d).

Next, we decided to determine the expression of all IL-6 family receptors including Interleukin-6 receptor (IL-6R), Interleukin-11 receptor (IL-11RA), IL-31RA, leukemia inhibitory factor receptor (LIFR), OSMR and its subunit IL6ST, ciliary neurotrophic factor receptor (CNTFR), that are widely known for their oncogenic actions^23^ in both sensitive and cisplatin-resistant ovarian cancer cells by qPCR (Fig. 1e). Importantly, OSMR is expressed highly in cisplatin-resistant OVCAR8 and A2780 cells included in GSE45553 and GSE33482 datasets (Supplementary Fig. 1g, h). Notably, we found that OSMR, LIFR, IL6R, and IL6ST mRNA were highly upregulated in cisplatin-resistant ovarian cancer cells in both A2780 and OVCAR8 cells (Fig. 1e). Because mRNA of OSMR family genes is highly expressed in cisplatin-resistant ovarian cancer and OSMR is the most highly expressed mRNA, we decided to determine OSMR protein level in a set of cisplatin resistant and sensitive form ovarian cancer cell lines. In consistent with mRNA data, protein levels of OSMR and its dimerizing partner IL6ST are highly upregulated in cisplatin-resistant A2780, OVCAR8, PEO4 and MCW-OV-SL3 ovarian cancer cell lines compared to their respective controls (Fig. 1f).

We further observed that the secreted levels of OSM, IL-6 and IL8 are high in supernatant of the cisplatin resistant cells, where OSM was the most upregulated cytokine (Supplementary Fig. 1i). Notably, we found that secreted OSM levels were higher than the secreted levels of IL-6 and IL-8, which are considered the most important proinflammatory interleukins produced by tumor cells.

Given that the receptors and ligands that activate the autocrine signaling through OSMR are highly expressed in cisplatin-resistant ovarian cancer cells, we sought to determine the level of OSMR/IL6ST heterodimerization in the cisplatin resistant cells compared to the sensitive cells. OSM binding to its receptor OSMR and heterodimerization with IL6ST is considered the first signaling event that is required for the initiation and activation of downstream signaling pathway such as JAK-STAT pathway. To examine the role of OSM on initiating OSMR-IL6ST heterodimerization, we performed a cross-linking dimerization assay in A2780 sensitive and A2780-CisR in response to OSM stimulation then treated with a membrane-impermeable chemical crosslinker BS3 (Fig. 1g). Our dimerization study using immunoprecipitation of OSMR antibody were able to capture OSMR-IL6ST dimerized receptor complex along with the monomer of OSMR in both cells, when treated with OSM. Notably, OSM-induced heterodimerization of OSMR was relatively high in A2780-CisR than A2780 sensitive, presumably due to high expression of OSMR and OSM in A2780-CisR cells (Fig. 1g). We further evaluated the endogenous phosphorylation levels of STAT3 in A2780 sensitive (parental) vs. A2780-CisR resistant and OVCAR8 sensitive (parental) vs. OVCAR8-CisR resistant cells and observed that phosphorylation of STAT3 (Y705, and S727 sites) were high in both A2780-CisR and OVCAR8-CisR cell lines (Fig. 1h).

### Overexpression of OSMR promotes chemoresistance in vitro

**Fig. 2:**
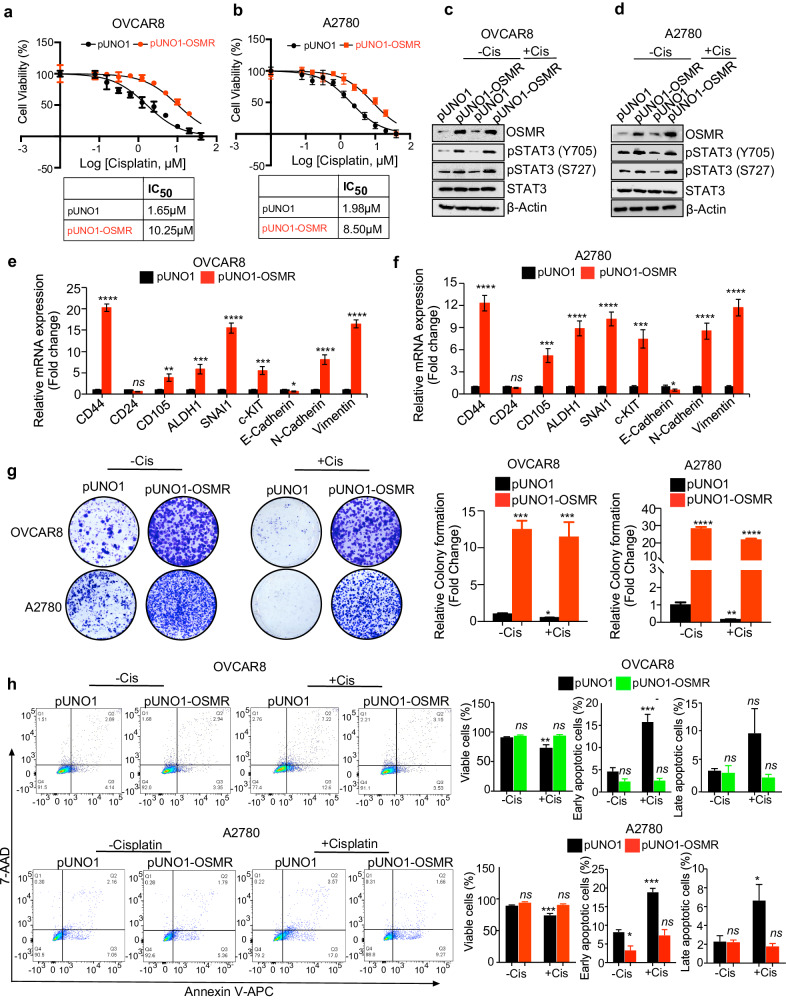
OSMR overexpression promotes chemoresistance. **a**, **b** Cell viability was determined using CCK8 reagent in both control and OSMR overexpressing OVCAR8 and A2780 cells were treated with different concentrations of cisplatin for 48 h. The IC_50_ of cisplatin in both control and OSMR overexpressing cell lines were quantitated and presented below in the respective boxes. **c**, **d** OVCAR8 and A2780 cells stably overexpressing OSMR, and the control cells were lysed and immunoblotted using indicated antibodies. **e**, **f** mRNA expression of cancer stemness markers and EMT markers were evaluated using qPCR in OVCAR8 and A2780 cells stably overexpressing OSMR and the control cells. **g** OVCAR8 and A2780 cells stably overexpressing OSMR and the control cells were grown in the presence and absence of cisplatin 5 μM for 15 days and imaged (left panel). Colonies formed were lysed using 10% acetic acid and absorbance were quantitated at 560 nm (right panel). **h** Control and OSMR overexpressing OVCAR8 and A2780 cells were treated with and without cisplatin for 16 h and the level of cellular apoptosis was quantitated using Annexin V-APC/7-AAD staining followed by flow cytometry (left). Bar graph shows percentage of viable cells, cells undergoing early, or late apoptosis quantitated from flow cytometry. Individual data points are represented as mean ± SEM. Student’s *t* test (two tailed, unpaired) was performed to determine significance between groups. ****P ≤ 0.0001, ***P ≤ 0.001, **P ≤ 0.01, *P ≤ 0.05, ns non-significant.

Next, we elucidated whether the overexpression of OSMR can induce chemoresistance in ovarian cancer cells, where we treated ovarian cancer cells with cisplatin and determined inhibitory concentrations of cisplatin in both sensitive and OSMR overexpressing cells using CCK8 cell viability assay. Notably, OSMR overexpression reduced the cisplatin sensitivity such that OSMR overexpression resulted in an increase in half maximal inhibitory concentration (IC_50_) of cisplatin from 1.65 μM to 10.25 μM and 1.98 μM to 8.50 μM in OVCAR8 and A2780 cells respectively (Fig. 2a, b).

Further, we examined the effect of OSMR overexpression on the downstream effectors such a STAT3 and how the treatment with cisplatin effects the phosphorylation and activation of STAT3. Immunoblotting assay in OVCAR8 and A2780 showed that OSMR overexpression significantly increased the phosphorylation of STAT3 (Y705 and S727) which is unchanged when treated with cisplatin (Fig. 2c, d).

Because EMT and cancer stemness are the key annotation marks in the cisplatin-resistant cells in our gene enrichment analysis (Fig. 1b and Supplementary Fig. 1d), we performed qPCR for EMT and cancer stemness-associated genes in OVCAR8 and A2780 sensitive cells to test if those markers are upregulated upon OSMR. As expected, we observed that the stable overexpression of OSMR increased the expression of EMT-associated genes (N-cadherin and vimentin) and cancer stemness-associated genes (CD44, SNAIL, and c-Kit) in both A2780 and OVCAR8 cells (Fig. 2e, f); whereas OSMR upregulation decreased the levels of CD24 and E-cadherin which are required for non-tumorigenic epithelial characteristics. Clonogenic assay further showed that cisplatin treatment in OSMR overexpressed OVCAR8 and A2780 cells did not make any profound impact on colony forming abilities of OVCAR8 and A2780 cells (Fig. 2g). Further we performed Annexin V-APC/7-AAD based apoptosis assay using flow cytometry of OSMR overexpressed OVCAR8 and A2780 cell lines that were treated with and without cisplatin. We also observed significant increase in early apoptosis in control cells, whereas a reduction in the number of apoptotic cells were observed in cisplatin treated OSMR overexpressing OVCAR8 and A2780 cells (Fig. 2h). Taken together, our results suggest that OSMR is critical modulator of cell survival, cancer stemness and EMT, and chemoresistance in ovarian cancer cells.

### Knockdown of OSMR inhibits cancer stemness and promotes cisplatin sensitivity in ovarian cancer cells in vitro

To further validate the impact of OSMR on cisplatin sensitivity, we stably knockdown OSMR using small hairpin RNA targets OSMR RNA in cisplatin-resistant OVCAR8 and A2780 cell lines which express high levels of OSMR and then treated with a range of concentrations of cisplatin and determined the IC_50_ of cisplatin in both control and OSMR knocked down cells using CCK8 cell viability assay. Notably, a significant level of reduction in the IC_50_ concentration of cisplatin was observed when OSMR was knockdown in both OVCAR8 and A2780 cell lines (Supplementary Fig. 2a, b).

We further examined the effect of OSMR knockdown on the downstream signaling molecule such as STAT3 and how the cisplatin treatment affects the phosphorylation of STAT3 (Y705 and S727), which is required for STAT3 transcriptional activity. Herein, our immunoblots showed that OSMR knockdown reduced the phosphorylation of STAT3 (Y705 and S727) compared to shControl cells (Supplementary Fig. 2c, d). Notably, we observed that cisplatin treatment in OSMR knockdown cells reduced the level of STAT3 phosphorylation (Y705 and S727) further effectively than OSMR knockdown alone (Supplementary Fig. 2c, d).

We also observed a significant reduction in EMT-associated genes and cancer stemness-associated genes and increase in E-Cadherin and epithelial cell marker CD24 when OSMR is knocked down in both OVCAR8-CisR and A2780-CisR resistant cells (Supplementary Fig. 2e, f). We next performed colony forming assay to determine if OSMR knockdown could sensitize the cisplatin resistant ovarian cancer cells to cisplatin therapy. While we noticed a decrease in colony formation in the cisplatin-resistant ovarian cancer cells, we also observed an improved level of inhibition in the colony forming ability of cisplatin resistant cells when OSMR was stably knockdown alone and in combination with cisplatin (Supplementary Fig. 2g, h). Next, we examined whether cisplatin treatment in OSMR knockdown cells can promote apoptosis using Annexin V-APC/7-AAD based apoptosis assay by flow cytometry. As expected, we observed significant increase in the number of early and late apoptotic cells in OSMR knockdown cell lines, which further improved when combined with cisplatin (Supplementary Fig. 2i, j). These data indicated that depleting OSMR by knockdown has a potential to resensitize ovarian cancer cells towards cisplatin therapy.

### OSMR signaling neutralization by anti-OSMR antibody promotes cisplatin resensitization and augments apoptosis

Recently, we have developed a panel of fully human anti-OSMR monoclonal antibodies using a phage-displayed single chain variable fragment (scFv) library by selecting scFv specifically bind the extracellular domain of OSMR and neutralize the signaling through OSMR protein^11^. Based on the data from the same study^11^, indicating that the anti-OSMR antibody clone named B21 clone was effective in inhibiting proliferation and inducing apoptosis of ovarian cancer cells both in vitro and in vivo, we sought to test the effect of this mAb clone on cisplatin sensitivity in both the sensitive and cisplatin-resistant ovarian cancer cells.

**Fig. 3:**
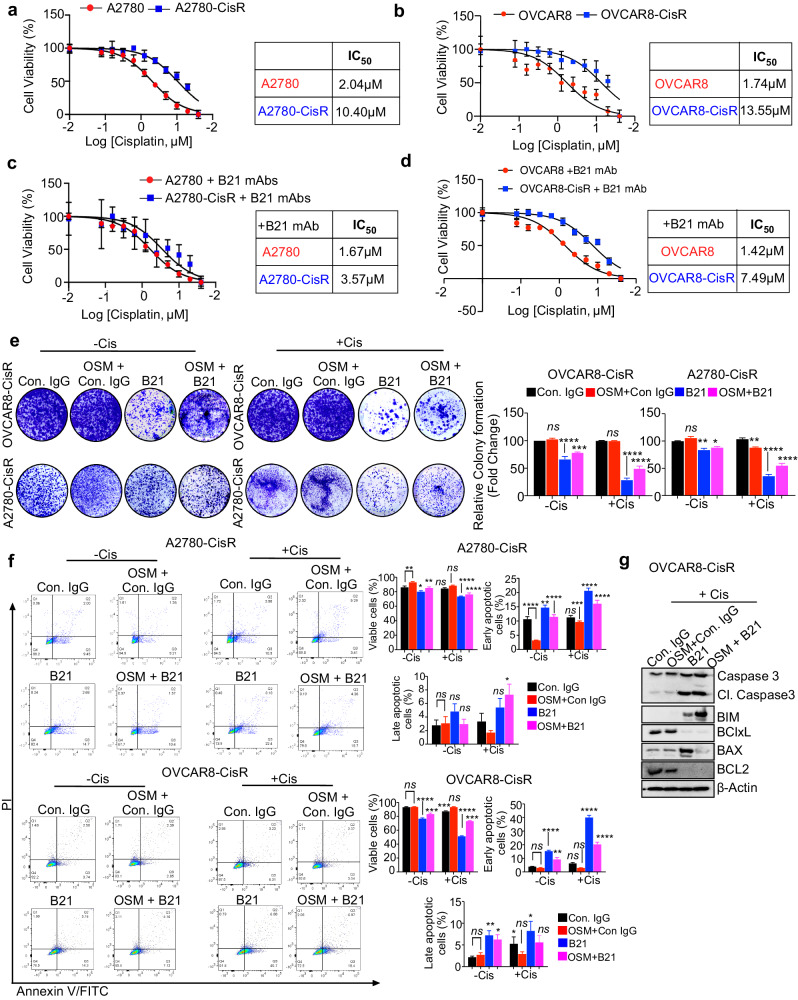
Anti-OSMR antibody enhances sensitivity towards cisplatin. **a**, **b** Cell viability was measured using CCK8 reagent in A2780 versus A2780-CisR cells, OVCAR8 versus OVCAR8-CisR cells were treated with different concentrations of cisplatin for 48 h. The IC_50_ of cisplatin in both control and OSMR overexpressing cell lines were quantitated and presented below in the respective boxes. **c**, **d** Cell viability assay was measured using CCK8 reagent in A2780 and A2780-CisR and OVCAR8 *vs*. OVCAR8-CisR resistant cells treated with anti-OSMR B21 mAb (10 μg/mL) in combination with the indicated concentrations of cisplatin for 48 h. The IC_50_ of B21 mAb in both control and OSMR overexpressing cell lines were quantitated and presented below in the respective boxes. **e** OVCAR8-CisR and A2780-CisR cells were grown in the presence and absence of B21 antibody or isotype control IgG (10 μg/mL each) with or without recombinant human rhOSM (100 ng/mL) for 15 days in 6-well plate. Colonies formed were stained using 0.5% of crystal violet and imaged. Colonies formed were lysed using 10% acetic acid and absorbance were quantitated at 560 nm (right panel). **f** Annexin V-FITC/PI based apoptosis assay was performed using flow cytometry in OVCAR8-CisR and A2780-CisR resistant cells that were treated with B21 antibody or isotype control IgG (10 μg/mL each) in combination with/without cisplatin and stimulated in the presence and absence of rhOSM (100 ng/mL) for 16 h. The representative quantitation histograms show percentage of cells that are viable and undergoing early or late apoptosis. **g** Western blot shows the expression of pro-apoptotic and anti-apoptotic proteins in OVCAR8-CisR cell lines that were treated with B21 anti-OSMR antibodies (10 µg/mL) in combination with cisplatin in the presence and absence of rhOSM (100 ng/mL) for 24 h. One-way ANOVA with Dunnett’s multiple comparison test was performed for significance. Data represent means ± SEM. ****P ≤ 0.0001, ***P ≤ 0.001, **P ≤ 0.01, *P ≤ 0.05, ns non-significant.

First, we tested the efficacy of anti-OSMR B21 mAb on the cell viability of A2780-CisR and OVCAR8-CisR resistant cell lines after treating with various concentrations of cisplatin. Notably, we observed a remarkable decrease in the IC_50_ of cisplatin in the cisplatin resistant cells when treated with anti-OSMR antibody. In brief, we observed ~69% decrease in IC_50_ in A2780 cells and ~50% decrease in IC_50_ in OVCAR8 cells (Fig. 3a–d). As expected, the cell viability of sensitive cell lines was relatively less, when treated with anti-OSMR antibody treatment as compared to the cisplatin-resistant cells, which express high levels of OSMR (Fig. 3a–d).

It has been reported that cancer stemness and EMT were associated with chemoresistance^24–27^. Since we observed that OSMR expression directly associates with cancer stemness and EMT markers (Fig. 2e), we investigated the effect of OSMR inhibition on cancer stemness in cisplatin-resistant ovarian cancer cell lines using colony formation and spheroid formation assays and found that the treatment of B21 anti-OSMR antibody reduced the colony forming abilities induced by OSM in both OVCAR8-CisR and A2780-CisR cell lines (Fig. 3e). We further investigated the effects of B21 antibody clone on cancer stemness and EMT by determining the level of markers associated with cancer stemness and EMT by qPCR and found that B21 antibody clone inhibited the expression of cancer stemness and EMT markers as compared to OSM-stimulated cells in OVCAR8-CisR and A2780-CisR resistant cells (Supplementary Fig. 3a, b). We also investigated the effects of anti-OSMR antibodies on the spheroid-forming ability of cisplatin-resistant cell lines by performing 3D-spheroid formation of A2780-CisR and OVCAR8-CisR on low-attachment plates for 15 days with B21 monoclonal antibody alone or in combination with cisplatin in the presence or absence of OSM. Notably, B21 treatment reduced the spheroid forming ability which was further reduced considerably when combined with cisplatin as compared to the control and OSM-stimulated cells (Supplementary Fig. 3c–f).

To further investigate the effect of anti-OSMR antibody on the downstream effectors of OSMR, we performed western blot analysis in OVCAR8-CisR resistant cells that were treated with B21 in combination with cisplatin in the presence and absence of OSM and found that the treatment with B21 mAb reduced phosphorylation of STAT3 at two key phosphorylation sites (S727 and Y705) significantly when treated in combination (Supplementary Fig. 3g). Taken together, our results suggests that anti-OSMR antibody in combination with cisplatin therapy are effective in inhibiting cancer stemness and sensitizing cisplatin. Therefore, OSMR targeting by neutralizing antibodies could be exploited to attenuate oncogenic signaling mechanisms associated with chemoresistance by treating with anti-OSMR antibodies for cisplatin sensitization.

Next, we validated the effect of combination treatment of B21 mAb with cisplatin on the apoptosis of cisplatin-resistant cell lines by performing Annexin V-FITC/PI staining followed by flow cytometry after blocking OSMR with B21 mAb. Markedly, A2780-CisR and OVCAR8-CisR cells treated with B21 mAb and in combination with cisplatin exhibited significantly high number of early and late apoptosis (Q3 and Q4 quadrants) (Fig. 3f). Based on our data that anti-OSMR antibodies were able to reduce cell viability and resensitize cisplatin therapy, we investigated the effects of combination treatment of B21 antibody clone with cisplatin on proteins which are important for apoptosis and found that anti-OSMR antibody B21 increased the expression of pro-apoptotic proteins cleaved caspase 3, and BAX and decreased the expression of anti-apoptotic proteins BCL2 and Bcl-xL in OVCAR8-CisR and A2780-CisR cell lines (Fig. 3g and Supplementary Fig. 3h).

Taken together, our results demonstrate that OSMR is an important mediator of cell survival and chemoresistance mechanism in ovarian cancer cells and inhibiting the oncogenic actions of OSMR using OSMR-specific antibody was able to sensitize cisplatin treatment.

### OSMR promotes the expression of a panel of integrins for cisplatin resistance through STAT3 transcription factor

**Fig. 4:**
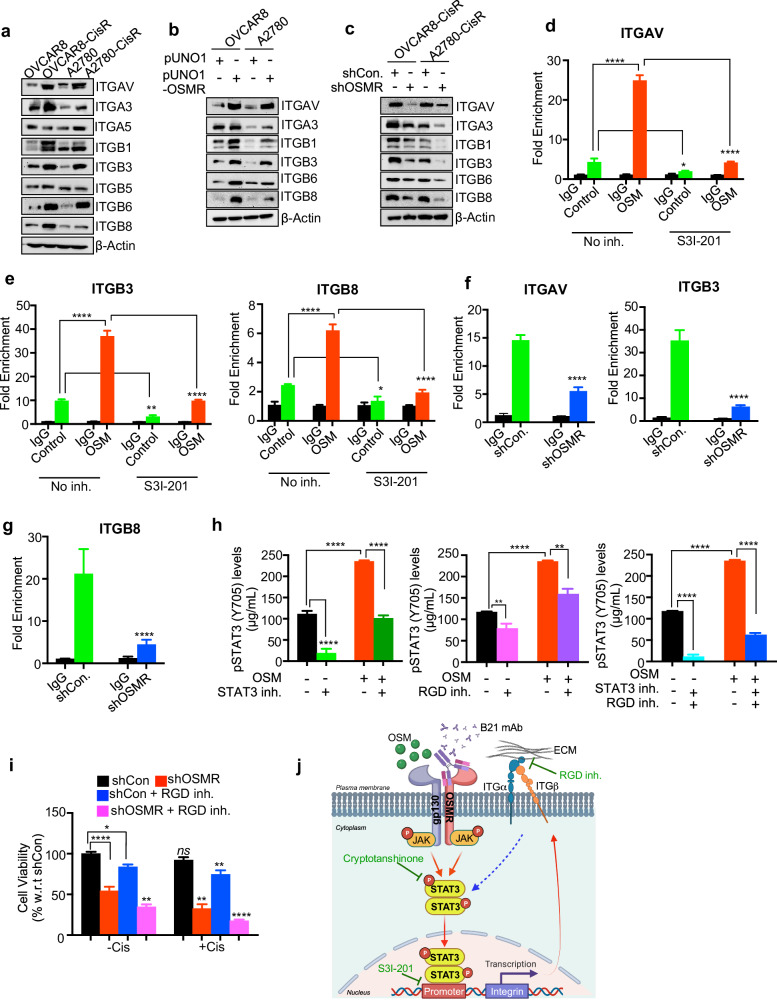
OSMR transcriptionally regulates integrins via STAT3 which facilitates OSMR signaling in promoting chemoresistance. **a** Western blot analysis shows the levels of integrins in OVCAR8, OVCAR8-CisR, A2780, A2780-CisR cells. **b** Western blot shows the level of selected integrins in both OVCAR8 and A2780 control cells and the cells stably overexpress OSMR. **c** OSMR was stably knocked down in cisplatin resistant OVCAR8 and A2780 cells and Western blot was performed using indicated antibodies specific to integrins. **d**, **e** OVCAR8 cells were stimulated in the presence and absence of rhOSM (100 ng/mL), with or without STAT3 inhibitor S3I-201 (50 μM) for 48 h. Chromatin-immunoprecipitation (ChIP) was performed using STAT3 antibody and qPCR was performed to determine the enrichment of promoters of ITGAV, ITGB3 and ITGB8 genes. The qPCR data are presented as fold enrichment compared to IgG control. **f**, **g** OSMR knocked down OVCAR8-CisR cells and empty vector expressing cells (shControl) were lysed, and chromatin-immunoprecipitation was performed using STAT3 antibody. Promoter enrichment of indicated genes were quantitated from the samples. **h** OVCAR8 cells were seeded on FN-precoated 96-well plates and treated with either STAT3 inhibitor, RGD peptide inhibitor or both for 30 min with or without rhOSM. Cells were lysed and pSTAT3 (Y705) levels were quantitated using ELISA. **i** OVCAR8-CisR cells stably knockdown with shOSMR were treated with RGD peptide inhibitor in the presence and absence of cisplatin for 48 h and cell viability assay was determined using CCK8. **j** Schema demonstrates how OSMR upregulates integrins through STAT3 activation and how does blocking of OSMR abrogates STAT3 mediated integrin levels. For significance, one-way ANOVA followed by Dunnett’s multiple comparison test were performed in ‘**h**’ and ‘**i**’ and Student’s *t* test (two tailed, unpaired) was performed for significance in ‘**d**, **e**’. Error bars represent means ± SEM. ****P ≤ 0.0001, ***P ≤ 0.001, **P ≤ 0.01, *P ≤ 0.05, ns: non-significant.

Our data identified that integrin signaling is the top enriched pathway among the genes upregulated in cisplatin-resistant cells (Fig. 1b). We further identified that several integrin family genes from the IPA analysis are positively correlated with OSMR (Supplementary Fig. 1e and Fig. 1c). Therefore, we sought to determine the role of integrins in promoting chemoresistance and characterize the mechanism of how integrin signaling is regulated by OSMR in cisplatin resistant ovarian cancer cells. First, we tested the expression of all the key α integrins such as ITGAV, ITGA3, ITGA5 and their respective dimerizing partner β subunits such as ITGB1, ITGB3, ITGB6, ITGB5, and ITGB8 in both cisplatin sensitive (parental) and OVCAR8-CisR and A2780-CisR resistant cell lines. Strikingly, we found that ITGAV, ITGA3, ITGB1, ITGB3, ITGB6, and ITGB8 are highly expressed in both mRNA and protein levels in cisplatin-resistant cell lines (Fig. 4a and Supplementary Fig. 4a). To further confirm the direct impact of OSMR on the above integrins, we have either overexpressed or knocked down OSMR in OVCAR8 and A2780 cell lines and determined the expression of ITGAV, ITGA3, ITGB1, ITGB3, ITGB6, and ITGB8. As expected, overexpression of OSMR significantly increased both mRNA and protein levels of ITGAV, ITGA3, ITGB1, ITGB3, ITGB6, and ITGB8 (Fig. 4b and Supplementary Fig. 4b). In contrast, OSMR knockdown reduced the expression of ITGAV, ITGA3, ITGB1, ITGB3, ITGB6, and ITGB8 in both mRNA and protein levels in OVCAR8 and A2780 cell lines (Fig. 4c and Supplementary Fig. 4c).

Next, we determined if STAT3 is required for OSMR-mediated transcription of integrins as key downstream processes. Herein, a chromatin immunoprecipitation (ChIP) assay was performed after the cells were stimulated with the ligand OSM that activates OSMR signaling in the presence or absence of STAT3 inhibitor of phosphorylation and promoter binding activity, named S3I-201 inhibitor and found that OSM stimulation enhanced the binding of STAT3 transcription factor on the promoters of ITGAV, ITGB3, and ITGB8 genes, which was abrogated when the cells were treated with S3I-201 (Fig. 4d, e and Supplementary Fig. 5a). To further confirm the role of OSMR, we stably knockdown OSMR using shOSMR in OVCAR8-CisR resistant cell lines and overexpressed OSMR in OVCAR8 cells and observed a significant reduction and enrichment in STAT3 binding on ITGAV, ITGB3, and ITGB8 promoter respectively (Fig. 4f, g and Supplementary Fig. 5b–d). In conjunction, we also observed a marked increase in the mRNA expression of ITGAV, ITGA3, ITGB1, ITGB3, ITGB6, and ITGB8 upon OSM stimulation, which was abolished upon the treatment of STAT3 inhibitor S3I-201 (Supplementary Fig. 4d). Furthermore, we also inhibited the OSMR-mediated signaling by blocking OSMR with anti-OSMR mAb B21 and found consistent results that blocking OSMR significantly reduced the mRNA expression of ITGAV, ITGA3, ITGB1, ITGB3, ITGB6, and ITGB8 (Supplementary Fig. 4e) and binding of STAT3 on ITGAV, ITGB3 and ITGB8 promoters (Supplementary Fig. 5e).

ITGAV, ITGB3, and ITGB8 are known as RGD receptor family, which requires the binding of integrin α and β subunits to ECM proteins containing the tripeptide Arg-Gly-Asp (RGD) domain such as fibronectin and vitronectin for integrin-mediated adhesion, migration, invasion, and subsequent intracellular signaling^18,19^. Therefore, we sought to examine the role of integrin interaction with fibronectin in conjunction with OSMR signaling for STAT3 activation and cell survival or proliferation. Herein, OVCAR8 cells were seeded on fibronectin-coated plates and treated with either STAT3 inhibitor, RGD peptide inhibitor, or their combination in the absence or presence of OSM. ELISA was performed to determine the STAT3-Y705 phosphorylation levels and found that the treatment with Cryptotanshinone, which inhibits STAT3 phosphorylation Y705, STAT3 dimerization and DNA binding significantly reduced OSM-induced STAT3 phosphorylation (Fig. 4h, left panel). Next, we compared the effect of STAT3 inhibitor with integrin-ECM interaction using an RGD peptide (GRGDNP) integrin interaction inhibitor on STAT3 phosphorylation. Notably, RGD peptide treatment was also able to reduce pSTAT3 levels, while the level of inhibition was modest compared to the STAT3 inhibitor (Fig. 4h, middle panel). These results suggest that the interaction of integrins with ECM is also an important mechanism that can activate STAT3 signaling. Importantly, we observed a synergistic inhibition on STAT3 phosphorylation when we combined both Cryptotanshinone and RGD inhibitors in the presence or absence of OSM stimulation (Fig. 4h, right panel), which suggest that blocking integrin signaling is important for sustained STAT3 signaling in ovarian cancer cells. We also determined the pSTAT3 levels in the cells were grown on fibronectin-coated plates after treating with anti-OSMR antibody and found that pSTAT3 levels was significantly reduced upon anti-OSMR antibody treatment when cells were grown in the presence of OSM as well as in the absence of OSM (Supplementary Fig. 4f). To validate further if OSMR is required for integrin-mediated cell survival, we used OSMR stably knockdown OVCAR8-CisR cell lines and plated on fibronectin-coated plates and treated with RGD inhibitor in combination with cisplatin and cell viability was evaluated. Herein, we observed that cisplatin treatment reduced cell viability in OSMR knockdown cells; whereas cisplatin treatment was not effective in the cells express control shRNA. We also observed that RGD blocking peptide treatment was effective on inhibiting cell viability modestly, RGD blocking was able to inhibit cell viability further when combined with cisplatin. Consistent with the above data, cisplatin treatment was more effective when the OSMR knockdown cells were treated along with RGD inhibitor (Fig. 4i). Taken together, our results demonstrates that OSMR-mediated upregulation of integrins contribute to STAT3 phosphorylation which is critical for cisplatin resistance (Fig. 4j).

### Delivery of anti-OSMR antibody sensitizes cisplatin treatment in ovarian cancer cells in vivo

**Fig. 5:**
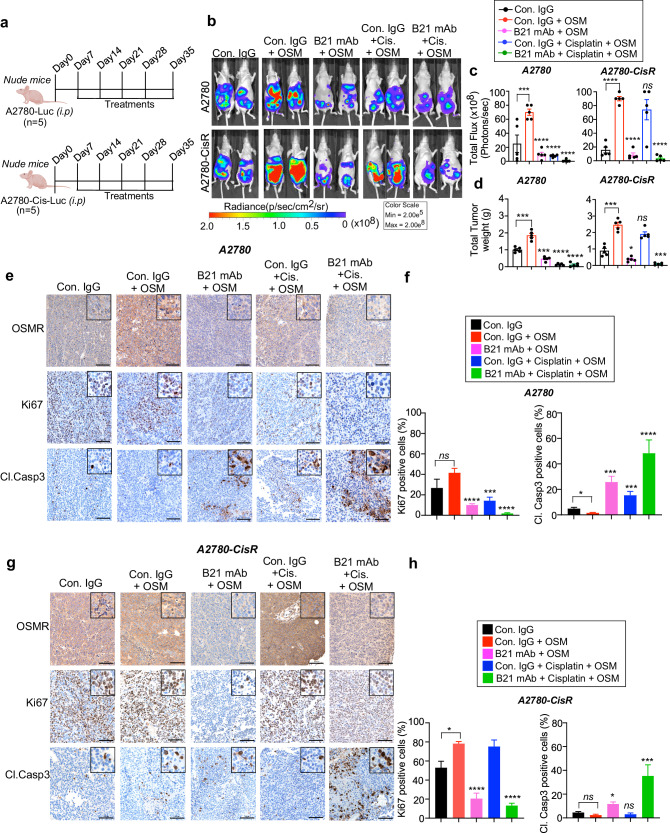
Anti-OSMR antibody increases the chemosensitivity of ovarian cancer cells towards Cisplatin in vivo. **a** Schema shows how A2780-Luc+ and A2780-CisR-Luc+ cells were grown in athymic nude mice and treated with isotype control IgG or B21 mAb (10 mg/kg body weight; i.p.) in the presence and absence of Cisplatin (5 mg/kg body weight) for 5 weeks. Mice were treated with OSM (250 ng/kg body weight) 30 minutes after the injections of control IgG, or B21 antibodies (10 mg/kg body weight) i.p. **b** Representative images showing rate of tumor growth was determined by bioluminescence imaging (BLI) using IVIS100 imager. **c** Quantitative assessment of luciferase signal intensity of A2780-Luc+ and A2780-CisR-Luc+ tumor bearing mice following the treatments before termination on day 35. **d** Total weight of the tumor in the respective treatment groups were quantitated. **e**, **g** Tumor tissues from A2780 and A2780-CisR bearing mice were subjected to immunohistochemistry using the indicated antibodies. Whole stained tissues sections were scanned, and representative images of the indicated proteins from each group were captured in ×20. Scale bar = 100 μm. **f**, **h** Quantitative assessment of Ki67 and cleaved caspase 3 cells in the respective treatment groups. One-way ANOVA with Dunnett’s multiple comparison test or Student’s *t* test were performed for determining significance. Data represent means ± SEM. ****P ≤ 0.0001, ***P ≤ 0.001, **P ≤ 0.01, *P ≤ 0.05. ns: non-significant.

Next, we confirmed the effect of anti-OSMR B21 mAb on inhibiting the growth of cisplatin-resistant ovarian cancer cells and sensitizing the efficacy of cisplatin in vivo. For this aim, we injected A2780 sensitive or A2780-CisR resistant cell lines stably expressing luciferase reporter intraperitoneally in athymic nude mice stimulated with OSM for OSMR signaling followed by control IgG or B21 antibody treatment (10 mg/kg body weight) in combination with cisplatin (5 mg/kg body weight) (Fig. 5a, b). Mice were then monitored for tumor burden by bioluminescence imaging (BLI) from days 7 to 35.

Monotherapy of B21 mAb or cisplatin considerably reduced the growth of A2780 ovarian cancer-bearing mice compared to the mice bearing A2780 ovarian cancer stimulated with OSM or control IgG (Fig. 5b, c). Markedly, the combination of B21 with cisplatin was able to reduce the tumor weight and tumor nodules considerably compared to the control (Fig. 5b–d). However, we did not find any significant difference in tumor growth when compared B21 treatment with cisplatin alone (Fig. 5b–d). Notably, mice injected with cisplatin-resistant A2780-CisR cells exhibited aggressive tumor growth pattern in control IgG and OSM-stimulated mice. Treatment of cisplatin alone did not inhibit the growth and metastasis of A2780-CisR tumors (Fig. 5b–d). While monotherapy of B21 mAb somewhat decreased the tumor growth of A2780-CisR bearing mice by day 35, the combination of B21 with cisplatin displayed significant reduction in the tumor growth, and number of tumor nodules in A2780-CisR bearing mice (Fig. 5b–d). Immunoblotting analysis using tumor tissues extracted from the above groups demonstrated a significant reduction of OSMR, and phosphorylated STAT3 levels in mice treated with B21 mAbs in combination with cisplatin in the A2780-CisR tumors (Supplementary Fig. 6a). Immunohistochemical analysis of the tumor tissues further showed a significant reduction in the OSMR and Ki67 levels and increase in Cleaved caspase 3 in B21 mAb alone, Cisplatin alone and in combination with B21 in A2780 bearing mice as compared to OSM-stimulated or control IgG treated (Fig. 5e, f). In cisplatin-resistant A2780-CisR bearing tumor, there was significant reduction in OSMR and Ki67 levels and increase in Cleaved caspase 3 levels when treated with B21 and cisplatin in combination (Fig. 5g, h). We further examined the expression of integrins ITGAV, ITGB3 and ITGA3 in the tumor samples by immunohistochemistry and found that integrin expression was significantly reduced in mice treated with B21 in combination with cisplatin at a higher level compared to monotherapy in A2780-CisR resistant cell line injected tumors (Supplementary Fig. 6b, c). Notably, we did not observe any significant changes in the weight of mice from all treatment conditions, indicating that there is no severe cytotoxicity upon anti-OSMR antibody treatment alone or in combination with cisplatin (Supplementary Fig. 6d). Taken together, our results demonstrate that inhibiting OSMR signaling using anti-OSMR antibody will sensitize cisplatin treatment, particularly when treating the cisplatin-resistant cells, which are often highly aggressive in nature.

## Discussion

Current standard treatment of advanced ovarian cancer patients involves neoadjuvant therapy and cytoreductive surgery followed by adjuvant chemotherapies such as platinum-based and taxane-based combination chemotherapies^28^. However, ∼50–70% of the ovarian cancer patients display recurrence due to chemoresistance. Although with the emergence of new FDA approved chemotherapeutic drugs such as PARP inhibitors (Olaparib, Niraparib), and VEGF inhibitors (bevacizumab), the survival outcome of patients has improved, yet the chemoresistance remains as a major challenge in the treatment of recurrent tumors.

Recent studies including ours have reported that Oncostatin M receptor (OSMR) is highly upregulated in aggressive cancers and is associated with unfavorable outcome in cancer patients^5–13^. LIFR serve as an alternative of OSMR for OSM when OSMR is expressed in low levels^29^. Notably, OSM interacts with OSMR and IL6ST dimers with high affinity and restrictively interacts with LIFR and IL6ST dimers. In contrast to LIFR, OSMR is highly expressed in fibroblasts, endothelial cells, and predominantly expressed in tumor cells compared to normal epithelial cells^11,30,31^. In the current study, we identified that OSMR is one of the highly upregulated genes in chemoresistant ovarian cancers particularly in the ovarian cancer cells expressing high levels of cancer stemness markers and EMT characteristics. We observed that OSMR is an important mediator for upregulating integrin expression for chemoresistance mechanism. We characterized that the crosstalk between OSMR and integrins through their interactions with respective ligands like OSM or fibronectin (FN) leads to the activation of STAT3 transcription factor for prolonged period in chemo resistant and highly aggressive ovarian cancer cells.

Based on our success on blocking OSMR signaling using monoclonal antibody abrogated STAT3 signaling repressed cancer stemness, 3-dimensional (3-D) spheroid forming ability, and ovarian cancer growth and progression^11^, we characterized the potential of testing the effect of anti-OSMR antibodies to treat cisplatin-resistant ovarian cancer cells. OSM has been widely known to promote cancer stemness and EMT in several cancers^8,32,33^. The role of OSM on resistance to chemotherapeutics such as etoposide and cisplatin has been initially reported in prostate cancer cells via IL6ST (gp130) signaling through MAPK and PI3K pathways^34^. It also has been reported that cancer stemness could be increased due to the activation of PI3K or JAK/STAT3 signaling^35^. In line with this notion, we provide evidence on OSM interaction with OSMR could be a trigger for resistance to drug-induced apoptosis through PI3K or MAPK pathway or STAT3 activation^5,11,36^. Subsequently, STAT3 activation resulted into the upregulation of integrins which in turn contributed into the STAT3 activation for a prolonged period as a critical mechanism in highly aggressive ovarian cancer cells like cisplatin-resistant cells.

Integrins are a large family of cell-surface transmembrane heterodimer receptors comprised of 18 α subunits and 8 β subunits and are classified into Arg-Gly-Asp (RGD) receptors that recognize RGD domain on the ECM ligands such as fibronectin, vitronectin (α5β1, α8β1, αVβ1, αVβ3, αVβ5, αVβ6, αVβ8, and αIIbβ3), collagen-binding integrin receptors (α1β1, α2β1, α10β1, and α11β1), laminin-binding integrins (α3β1, α6β1, α7β1, and α6β4), and leukocyte-integrins (α4β1, α9β1, α4β7, αEβ7,αLβ2, αMβ2, αXβ2, and αDβ2)^17–19^. Several studies show that integrins are expressed at high levels in various cancer types including ovarian cancer is often associated with poor prognosis and chemoresistance^18,37,38^. Using clinical datasets and in vitro approaches, we found that integrin dimers such as αVβ1, αVβ3, αVβ6, αVβ8, or α3β1 could feed into the survival signaling mechanisms in cisplatin-resistant cancer cells. Extensive studies including ours have reported that integrins expressed on tumor surface interacts with the ECM in tumor microenvironment (stroma) and contribute to critical oncogenic functions such as migration, invasion, adhesion, angiogenesis, and peritoneal spreading of cancer cells^39,40^.

Our ChIP further characterized that OSMR-mediated STAT3 activation resulted into the transcription of all the key integrins such as αV, β3, and β8 integrins. Interestingly, we also observed that STAT3 phosphorylation levels are regulated not only by OSM/OSMR interaction, but also by integrin/ECM interaction which suggests a crosstalk between integrins and OSMR signaling. Several studies have documented that integrins for example, αvβ3, α6β4, αvβ6 crosstalk with cell-surface receptors such as EGFR, IGF1, ERBB2, respectively, to promote tumorigenesis^18,41–43^. We observed that abrogating the interaction of RGD-binding integrins with fibronectin, considerably reduced the phosphorylation of STAT3, however not completely, suggesting that the cooperation between OSM/OSMR pathway with integrin signaling functioning as an additive signaling mechanisms in cisplatin-resistant cancer cells.

We have published that the integrin signaling could activate ZEB1 transcriptional repressor^39^, which could be critical features of cancer stem cells (CSC) and epithelial to mesenchymal transition (EMT) are often associate with chemoresistance. Ovarian CSCs express high CD44+and CD117+ (a.k.a. c-KIT) are known for chemoresistance to cisplatin and paclitaxel^25,44,45^. Integrin signaling has also been found to play a crucial role in cancer stemness by activating or regulating several signaling pathways and proteins, thus modulating the interaction between cancer cells and ECM. For instance, αvβ5 integrin-mediated signaling enhances the STAT3 (Y705) phosphorylation to expand CSCs population in pancreatic cancer^46^. Studies were also reported that α6β4 integrin mediated SRC/β-catenin signaling enhances cancer stemness properties by upregulating CD44 and EGFR^47^. Several studies have reported that integrins such as αvβ3 promote resistance to chemotherapy, molecularly targeted therapy and radiotherapy in several cancers including ovarian cancer by overcoming anoikis, autophagy, and apoptosis by increasing p27 and Bcl-2 levels and inhibition of caspase function^48–51^

Taken together, our data suggest that OSMR is an important therapeutic liability in ovarian cancer cells, particularly in cisplatin resistant ovarian cancer cells. Therefore, blocking OSMR signaling using the monoclonal antibodies that specifically binds and inhibits the signaling mechanisms could be a viable strategy to abrogate chemoresistance by reducing the integrin levels, and interaction between integrins and ECM. Immunotherapy is revolutionizing the current treatment strategies of several cancer including ovarian cancer. Several immunotherapy treatment modalities both as monotherapy and combination therapies with standard treatments are currently being tested under clinical trials such as targeted antibody-drug conjugates (ADCs), Bi-specific T cell-engaging antibodies (BiTEs) (targeting Angiopoietin, RANKL, CA125, CD274/PDL1, HER2. B7-H3)^52^, and chimeric antigen receptor T (CAR-T) cell therapies^53^. Towards the goal of treating ovarian cancer patients, we have developed monoclonal antibodies targeting the extracellular domain of OSMR^11^.

We found that our anti-OSMR monoclonal antibodies reduced the ovarian cancer cell growth and progression by inhibiting STAT3 activation and subsequent oncogenic signaling operated through STAT3, inhibiting the levels of survival regulators such as BCl2, PCNA, and inducing cell death. We observed that our antibodies reduced the cell viability of cisplatin-resistant ovarian cancer cells considerably which was relying on OSMR signaling and therefore showed sensitization towards cisplatin therapy both in vitro and in vivo. Taken together, our findings have provided new insights on the role of OSM-OSMR signaling in promoting cisplatin resistance in ovarian cancer and opportunities to reverse cisplatin-resistant mechanisms in ovarian cancer through targeting anti-OSMR antibodies.

## Methods

### Cell lines and cell culture

A2780 cell line was purchased from NCI-DCTD tumor repository and A2780 cisplatin-resistant (A2780-CisR) human ovarian cancer cell lines was purchased from European Collection of Authenticated Cell Cultures (ECACC)-Sigma Aldrich. PE01 (BRCA2-deficient, platinum sensitive), and PE04 (BRCA2-proficient, platinum resistant) were kindly provided by Daniela E Matei, Northwestern University, Chicago, Illinois, USA. OVCAR8 cell line was purchased from National Cancer Institute (NCI). OVCAR8 cisplatin-resistant (OVCAR8-CisR) cells were developed from OVCAR8 parental cell line by continuous exposure to increasing dose of cisplatin (Selleckchem, TX, USA) for a period of 12 months. MCW-OV-SL-3 ovarian cancer cell line was established and characterized in our laboratory as described previously^54^. To maintain cisplatin resistance, the cisplatin-resistant cell lines were cultured in 1 μM cisplatin as per supplier’s guidelines. All cell lines were cultured in Dulbecco’s modified Eagle’s medium (DMEM) (Sigma Aldrich, MO, USA) containing glucose and 4 mM L-glutamine (Thermo Fisher Scientific, MA, USA) with 10% fetal bovine serum (Omega Scientific, CA, USA), and supplemented with 1% Anti-anti antibiotic (Thermo Fisher Scientific, MA, USA) at 37 °C in a humidified incubator with 5% CO_2_ as described previously^54^. Each cell line was authenticated by short tandem repeat (STR) profiling (IDEXX BioAnalytics) and tested for Mycoplasma using MycoSensor PCR Assay kit (Agilent, Santa Clara, CA).

### Cell transfection

The stable knockdown of shOSMR was performed as described earlier^11^. pLKO.1-shOSMR expression plasmids (MISSION® pLKO.1-puro-shOSMR #1 TRCN0000058687, Clone ID: NM_003999.1-2459s1c1 5′CCGGGCATTGATTGTGGACAACCTACTCGAGTAGGTTGTCCACAATCAATGCTTTTTG3′) targeting the coding region of human OSMR and human MISSION® pLKO.1-puro Empty Vector control plasmid (Cat# SHC001V) were purchased from Sigma Aldrich (Saint Louis, MO). Lentiviral particles of the respective vectors were prepared in Viral Vector Core Lab (Versiti) and were transduced in OVCAR8-CisR and A2780-CisR cells with polybrene (8 μg/mL) and Puromycin (Thermo Fisher Scientific, MA, USA) (final concentration = 8 μg/mL) was used to select infected cells.

Stable overexpression cell lines, pUNO1-OSMR vector (Invivogen, CA, USA) targeting the coding region of human OSMR, and non-specific control pUNO1-Control vector (Invivogen, CA, USA) were prepared as described previously^11^. Briefly, pUNO1-OSMR and pUNO1-Control plasmids were transfected in OVCAR8 and A2780 cell lines using Lipofectamine 2000 (Thermo Fisher Scientific, MA, USA) and selection antibiotic Blasticidin (Thermo Fisher Scientific, MA, USA) (final concentration = 10 μg/mL) were used to select infected cells according to manufacturer’s guidelines.

### Antibodies

The antibodies and reagents used in this study are listed in Supplementary Table S1. Anti-OSMR antibodies were developed by panning a large human scFv phage display antibody library as described previously^11^.

### Animal studies

All animal work was done in accordance with protocol approved by the Institutional Animal Care and Use Committee (IACUC) at the Medical College of Wisconsin. To evaluate the in vivo Cisplatin drug sensitivity by anti-OSMR antibody, A2780-Luc+ cells and A2780-CisR-Luc+ cells (1 × 10^6^ cells/animal) were intraperitoneally injected into 4-6-week-old athymic female nude mice (Nu/Nu) (Envigo, Madison, WI, USA) with a 27-gauge needle. On Day 7, mice were pre-anesthetized using isoflurane chamber and injected intraperitoneally (IP) with 200 μL D-Luciferin (GoldBio, 15 mg/mL in PBS) and then transferred to the IVIS 100 analyzer for bio-luminescence imaging (BLI) under isoflurane anesthesia. Based on approximately uniform tumor distribution, mice were randomly divided into subgroups (*N* = 5/group) and imaged once *weekly* using IVIS 100 imager for 5 weeks following respective treatments. Subsequently, mice were treated intraperitoneally twice weekly with Group1: Control IgG (10 mg/kg b.w), Group 2: Control IgG(10 mg/kg b.w) + recombinant human rhOSM (250 ng/kg b.w) (R&D Systems, USA), Group 3: B21 mAb (10 mg/kg b.w) + rhOSM (250 ng/kg b.w), Group 4: Control IgG (10 mg/kg b.w) + Cisplatin (5 mg/kg b.w) + rhOSM (250 ng/kg b.w), Group 5: B21 mAbs (10 mg/kg b.w) + Cisplatin (5 mg/kg b.w) + rhOSM (250 ng/kg b.w) in 100 μL PBS for a total of 5 weeks. All mice were euthanized with CO_2_ exposure (30–70% vol/min flow rate) inside a cage followed by cervical dislocation at the end of 5 weeks. Tumors were harvested, weighed, and excised to be proceeded for IHC and Western blotting.

### Fibronectin coating

Fibronectin (FN) (Sigma Aldrich, MO, USA) is prepared at a working concentration of 10 μg/ml in PBS and 30 μl per well is coated in each well of 96-well plate and incubated at 37 °C, 5% CO_2_ incubator for 60 min^55^. Excess FN was washed gently twice with PBS. To block chemoattractant present in ECM proteins, 50 μl of heat denatured BSA (10 mg/mL) (Sigma Aldrich, MO, USA) into each well and incubated for 30 min at room temperature, followed by washing with PBS.

### ELISA

For Multiplex ELISA, the levels of endogenous OSM, IL31, LIF, IL-6, and IL-8 were measured using custom-made Luminex Multiplex ELISA (R&D Systems) in the culture supernatants of A2780-CisR *vs*. sensitive cell lines according to manufacturer’s guidelines. 100 μL cell supernatant was added to a mixture of color-coded magnetic beads, pre-coated with cytokine-specific capture antibodies. The antibodies bind to the cytokines of interest present in culture supernatants. Biotinylated detection antibodies specific to the cytokines of interest are added which form an antibody-antigen sandwich. Phycoerythrin (PE)-conjugated streptavidin was further added which binds to the biotinylated detection antibodies. The magnetic bead-antibody complexes were detected on a dual-laser flow-based Bio-Plex®200 (Bio-Rad) Analyzer.

For pSTAT3 (Y705) ELISA, the cells (8 × 10^3^ cells/well) were seeded on 96-well FN-precoated plates. Following adherence, the cells were treated with Cryptotanshinone (Selleckchem, TX, USA) (10 μM) or RGD peptide (GRGDNP) (200 μM) inhibitor (Selleckchem, TX, USA) for 30 min followed by stimulation with OSM (100 ng/mL) for 30 min. The cells were washed with PBS and harvested using cell lysis buffer. pSTAT3 levels were determined in the lysates using PathScan® Phospho-Stat3 (Tyr 705) Sandwich ELISA Kit (Cell Signaling Technologies, MA, USA) as per manufacturer’s instructions.

### 3-Dimensional culture of tumor cells

Tumor cells were cultured as 3-D spheroids as described previously^11^. Cells were harvested, counted, and seeded onto ultra-low attachment 24-well Culture plates and suspended in 1:1 ClonaCell medium (StemCell Technologies, Vancouver, Canada): DMEM. The seeded cells and formed spheres were cultured in complete medium up to day 15. The viability of spheroids was measured using CellTiter-Glo® 3D Cell Viability Assay (Promega Corporation, WI, USA) according to manufacturer’s guidelines. The size of spheroids was measured using NIS Essentials software (Nikon Instruments, Melville, NY).

### Clonogenic assay

Colony formation assay was performed as mentioned previously^5,56^ with some modification. Briefly, the cells were seeded into six-well plates at 1000 cells per well with complete growth medium 24 h prior to treatment and cultured for 15 days. On the tenth day, the colonies were fixed with 5% gluteraldehyde and stained with 0.5% crystal violet. Plates containing the colonies were washed with water and dried before imaging. Colonies were solubilized with 10% acetic acid and quantified by reading absorbance at 560 nm.

### Cell viability assay

Cells (8 ×10^3^ cells/well) were seeded in 96-well plates or in FN-precoated 96-well plate. Following treatment for 48 h, the cells were incubated for 3 h with CCK8 reagent at 37 °C in 5% CO_2_ incubator according to manufacturer’s guidelines. The absorbance was measured at 450 nm.

### Flow Cytometry Analysis

Ovarian cancer cells were seeded in 100 mm^3^ dish (1 × 10^6^ cells/well) and were treated with control IgG, rhOSM (100 ng/mL), and B21 (10 μg/mL) without and with cisplatin (5 μM: A2780-CisR and 10 μM: OVCAR8-CisR) for 16 h. A2780 and OVCAR8 stably overexpressing OSMR and cells A2780-CisR and OVCAR8-CisR with stable knockdown of OSMR were seeded in 100 mm^3^ dish (1 × 10^6^ cells/well) and treated with cisplatin (5 μM: A2780; 5 μM: OVCAR8) for 16 h. Apoptosis assay by flow cytometry was performed as previously described^39^. Briefly, the cells were washed three times with PBS, harvested by trypsin, and subsequently stained with Annexin V-FITC or Annexin-V-APC and propidium iodide or 7-AAD (Biolegend, CA, USA) as per manufacturer’s guidelines, and subjected to flow cytometry (BD Biosciences) to detect apoptosis. The percentages of cells that were Annexin V^-^PI^-^/7-AAD^-^ (viable cells), Annexin V^+^PI^-^/7-AAD^-^(early apoptotic), Annexin V^+^PI^+^/7-AAD^+^ (late apoptotic) cells were analyzed by FlowJo software (BD Bioscience).

### Western blot analysis

Cells were washed twice in ice-cold PBS and lysed in RIPA buffer supplemented with protease inhibitor cocktail (Santa Cruz Biotechnologies) as mentioned previously^57^. Total protein concentrations were determined using BCA kit (Thermo Fisher Scientific) and 30μg protein was separated by 8% or 10% SDS-PAGE and transferred onto PVDF membranes (Bio-Rad, Hercules, CA). After blocking with 5% non-fat milk (Bio-Rad, Hercules, CA), membranes were incubated with indicated primary antibodies (Supplementary Table 1) overnight at 4 °C and corresponding HRP-conjugated secondary antibodies (Cell Signaling Technologies, MA, USA). Immunoreactive signals were detected using chemiluminescence detection kit (Bio-Rad, Hercules, CA). Un-cropped form of all Western blots are presented in Supplementary Fig. 7.

### Dimerization assay

Dimerization assay was performed as mentioned previously^11^. A2780 and A2780-CisR cells were seeded overnight and upon 70–80% confluency, serum starved for 6 h and stimulated in the presence of OSM (100 ng/mL) for 60 min on ice to prevent internalization of dimerized receptors. The cell lysates were incubated with non-permeable cross-linking reagent, 3 mM bis (sulphosuccinimidyl) suberate [BS3(Pierce)], cross-linking reagent on ice for 30 min and subsequently quenched with 250 mM Glycine. The cells were washed with ice-cold PBS and lysed using RIPA buffer as mentioned earlier. The pre-cleared lysates were immunoprecipitated overnight at 4 °C using anti-OSMR antibody bound to Dynabeads (Thermo Fisher Scientific) and eluted using 1x Laemmli sample buffer and the proteins were separated on a 6% SDS/PAGE. Separated proteins were transferred to PVDF membrane and immunoblotted with OSMR, IL6ST antibodies.

### Immunohistochemistry

The tumor tissues from A2780-Luc+ and A2780-CisR-Luc+ injected mice were cut into thin slices and fixed in 1% neutral formalin jars for 72 h. The slices were paraffin embedded and sectioned (4μm thickness) in Children’s Research Institute (CRI) Histology Core. Immunohistochemistry was performed as previously described^58^ with some modifications. Briefly the tumor tissue sections were baked at 60 °C for 60 min, deparaffinized in xylene and hydrated in varying alcohol concentrations (100% to 70%). Antigen retrieval was performed for 40 min in a steamer using IHC Tek solution. The sections were subsequently blocked in Normal Horse serum (Vector Laboratories, CA, USA) for 60 min and incubated with respective primary antibodies (Supplementary Table 1) overnight at 4 °C. The slides were incubated with ImmPRESS HRP Horse Anti-Rabbit or Anti-Mouse IgG PLUS Polymer Kit, Peroxidase secondary antibody (Vector Laboratories, CA, USA) and developed using ImmPACT® DAB Substrate Kit, Peroxidase (HRP) (Vector Laboratories, CA, USA). The sections were counterstained with Gill’s hematoxylin (Vector Laboratories, CA, USA) and Scott’s tap water substitute was used for bluing. The sections were dehydrated and mounted in DPX (Sigma). Stained whole tissue sections were scanned using Pannoramic 250 scanner (3D Histech, Hungary) and images were captured in 20x.

### RNA isolation, qRT-PCR, and qRT-PCR array

Total RNA was extracted using Qiagen RNeasy Plus Mini Kit (Qiagen, Frederick, MD., USA) as described previously^57^. RNA (1 µg) was reverse transcribed using iScript Reverse Transcription Supermix (Bio-Rad). qRT-PCR was performed with iTaq Universal SYBR Green Supermix (Bio-Rad) according to manufacturer’s instructions using a Bio-Rad CFX Connect Real Time PCR system (Bio-Rad). Each reaction was performed in triplicate. mRNA expression was normalized to β-Actin by 2^-ΔΔCt^. Primer sequences used in this study are listed in Supplementary Table S2.

Relative expression of 84 genes involved in IL6 family of ligands and receptors and IL6 signaling pathway was analyzed in A2780-CisR resistant and A2780 sensitive cells using Human IL-6 Signaling Pathway RT^2^ Profiler™ PCR Array (Cat#330231, PAHS-160ZA, Qiagen, Frederick, MD., USA). Total RNA was isolated from both cell lines using Qiagen RNeasy Mini Kit by following manufacturer’s protocol and quantified by a Nanodrop 2000. cDNA was synthesized, and qRT-PCR was performed in technical triplicates in 96-well PCR array plates on BioRad CFX Connect. Each array contained five separate housekeeping genes (B2M, ACTB, GAPDH, HPRT1, and RPLP0) that were used for normalization of the sample data. Raw data from the qRT-PCR was uploaded in GeneGlobe Data Analysis Center (https://geneglobe.qiagen.com/us/analyze/) and fold changes of gene expression, and volcano plot relative to A2780 sensitive cells were generated using VolcaNoseR^59^ and were considered ≥ 1.5-fold change as upregulated and ≤0.67 as downregulated with P ≤ 0.05. For these calculations, based on lower % CV of housekeeping genes, the average expression of the reference gene B2M was used for normalization of the data.

### Chromatin Immunoprecipitation assay

The ChIP assay was performed as outlined previously^39^ with some modifications using SimpleChIP® Enzymatic Chromatin IP Kit (Magnetic Beads) (Cell Signaling Technologies, #9003). In brief, cells were seeded in 150 mm dish and after treatment with inhibitors or stimulated with OSM for 48 h, the cells were crosslinked by adding 37% formaldehyde to a final concentration of 1% for 15 min at room temperature followed by quenching with glycine. Subsequently, cells were washed with PBS and subjected to cell lysis using cell lysis buffer provided in the kit. The cell extracts were sonicated using Bioruptor at 4 °C and DNA was extracted and ran on 2% agarose gel to check the appropriate shearing. The lysates were immunoprecipitated with STAT3 antibody (Cell Signaling Technology) and DNA was isolated and purified. DNA obtained was quantitated using Tecan and qPCR was performed using the designed promoter primers. The sequence of promoter primers is listed in Supplementary Table S3. ChIP data was analyzed as fold enrichment w.r.t. the IgG controls. The promoter sequences of ITGAV, ITGB3, and ITGB8 were obtained from CiiDER and TFBind and respective primers were designed using iDT primer designing tool.

### Statistical analysis

Cell culture-based experiments were repeated at least three times (three biological replicates) and all data were expressed as means ± SE. All statistical analysis were performed in GraphPad Prism (v 9.0). For all in vitro experiments, three or six technical replicates were analyzed for each experiment, and results are presented as the mean ± S.E.M. of three biological replicates. Quantitative analyses were carried out using two-tailed Student’s *t* test with standard error. For all RT-qPCR experiments, three technical replicates were analyzed for each experiment, and results are presented as the mean ± S.E.M. of three biological replicates. Statistical analyses of in vitro and in vivo experiments with four or more groups were performed with one-way ANOVA followed by Dunnett’s multiple comparison test. *P* values < 0.05 (*), < 0.01 (**), < 0.001 (***), and <0.0001 (****) were considered as statistically significant.

### Reporting summary

Further information on research design is available in the Nature Research Reporting Summary linked to this article.

### Supplementary information

Combined supplementary materials

REPORTING SUMMARY

## Data Availability

The authors declare that all data supporting the findings of this study are available within the paper and the supplementary file linked to this article. Any additional information, resources and reagents related to this paper is available from the corresponding authors upon request. The materials and data will be made available upon request after the completion of a material transfer agreement.

## Code availability

Gene expression microarray profiling data, GSE45553 and GSE33482, were obtained online from Gene Expression Omnibus (http://www.ncbi.nlm.nih.gov/geo). Differentially expressed genes were identified by DESeq2 using R software. Analysis was conducted following a standard four-step approach, cleaning, aligning, counting, and differential expression with an additional normalization step. Genes that were above 1.5-fold (log2 fold change ≥ 0.58) and below 0.67-fold (log2 fold change ≤ -0.58) were considered upregulated and downregulated respectively with significance *P* value adjusted FDR < 0.1. Pathway analysis of significantly differentially expressed genes was conducted using Ingenuity Pathway Analysis (IPA) to identify the canonical pathways. Volcano plot was prepared using VolcaNoseR^59^. Spearman’s correlation analysis was performed to analyze the correlation between OSMR and integrin family genes from TCGA-OV dataset consisting of 379 Ovarian Serous Cystadenocarcinoma patient cohort was downloaded from GDC data portal (https://portal.gdc.cancer.gov) via Bioconductor package TCGA and gene expression profiles were processed via HTSeq pipeline. Correlation plots of Integrin signaling pathway genes and integrin genes with OSMR from the microarray dataset (GSE45553) were plotted using R software where *P* value of Spearman’s correlation is ≤0.05. Venn diagram was prepared using Venny 2.1.0.

## References

[1] Marcus CS, Maxwell GL, Darcy KM, Hamilton CA, McGuire WP (2014). Current approaches and challenges in managing and monitoring treatment response in ovarian cancer. J. Cancer.

[2] Wang Y (2010). Autocrine production of interleukin-6 confers cisplatin and paclitaxel resistance in ovarian cancer cells. Cancer Lett..

[3] Wang, Y. et al. IL-6 mediates platinum-induced enrichment of ovarian cancer stem cells. *JCI Insight*10.1172/jci.insight.122360 (2018). 10.1172/jci.insight.122360 PMC6328027 30518684

[4] Masjedi A (2021). Oncostatin M: A mysterious cytokine in cancers. Int. Immunopharmacol..

[5] Parashar D (2019). miRNA551b-3p activates an oncostatin signaling module for the progression of triple-negative breast cancer. Cell Rep..

[6] Araujo, A. M. et al. Stromal oncostatin M cytokine promotes breast cancer progression by reprogramming the tumor microenvironment. *J. Clin. Invest.*10.1172/JCI165107 (2022). 10.1172/JCI165107 PMC9525111 36169029

[7] Lee BY (2021). Heterocellular OSM-OSMR signalling reprograms fibroblasts to promote pancreatic cancer growth and metastasis. Nat. Commun..

[8] Smigiel JM, Parameswaran N, Jackson MW (2017). Potent EMT and CSC phenotypes are induced by oncostatin-M in pancreatic cancer. Mol. Cancer Res..

[9] Zhu M (2015). Oncostatin M activates STAT3 to promote endometrial cancer invasion and angiogenesis. Oncol. Rep..

[10] Kucia-Tran JA (2016). Overexpression of the oncostatin-M receptor in cervical squamous cell carcinoma is associated with epithelial-mesenchymal transition and poor overall survival. Br. J. Cancer.

[11] Geethadevi A (2021). Oncostatin M receptor-targeted antibodies suppress STAT3 signaling and inhibit ovarian cancer growth. Cancer Res..

[12] Junk DJ (2017). Oncostatin M promotes cancer cell plasticity through cooperative STAT3-SMAD3 signaling. Oncogene.

[13] Wallace PM (1999). Regulation of inflammatory responses by oncostatin M. J. Immunol..

[14] Di Maira G (2022). Oncostatin M is overexpressed in NASH-related hepatocellular carcinoma and promotes cancer cell invasiveness and angiogenesis. J. Pathol..

[15] West NR, Murphy LC, Watson PH (2012). Oncostatin M suppresses oestrogen receptor-alpha expression and is associated with poor outcome in human breast cancer. Endocr. Relat. Cancer.

[16] Verstockt S (2021). Oncostatin M is a biomarker of diagnosis, worse disease prognosis, and therapeutic nonresponse in inflammatory bowel disease. Inflamm. Bowel Dis..

[17] Li S, Sampson C, Liu C, Piao HL, Liu HX (2023). Integrin signaling in cancer: bidirectional mechanisms and therapeutic opportunities. Cell Commun. Signal..

[18] Liu F, Wu Q, Dong Z, Liu K (2023). Integrins in cancer: emerging mechanisms and therapeutic opportunities. Pharm. Ther..

[19] Takada Y, Ye X, Simon S (2007). The integrins. Genome Biol..

[20] Dijkgraaf EM, Welters MJ, Nortier JW, van der Burg SH, Kroep JR (2012). Interleukin-6/interleukin-6 receptor pathway as a new therapy target in epithelial ovarian cancer. Curr. Pharm. Des..

[21] Duan Z, Feller AJ, Penson RT, Chabner BA, Seiden MV (1999). Discovery of differentially expressed genes associated with paclitaxel resistance using cDNA array technology: analysis of interleukin (IL) 6, IL-8, and monocyte chemotactic protein 1 in the paclitaxel-resistant phenotype. Clin. Cancer Res..

[22] Chowanadisai, W. et al. Cisplatin resistant spheroids model clinically relevant survival mechanisms in ovarian tumors. *PLoS ONE*10.1371/journal.pone.0151089 (2016). 10.1371/journal.pone.0151089 PMC4795743 26986722

[23] Heinrich PC (2003). Principles of interleukin (IL)-6-type cytokine signalling and its regulation. Biochem. J..

[24] Dar S (2017). Bioenergetic adaptations in chemoresistant ovarian cancer cells. Sci. Rep..

[25] Chau WK, Ip CK, Mak AS, Lai HC, Wong AS (2013). c-Kit mediates chemoresistance and tumor-initiating capacity of ovarian cancer cells through activation of Wnt/beta-catenin-ATP-binding cassette G2 signaling. Oncogene.

[26] Phi LTH (2018). Cancer stem cells (CSCs) in drug resistance and their therapeutic implications in cancer treatment. Stem Cells Int..

[27] Li Y, Wang Z, Ajani JA, Song S (2021). Drug resistance and cancer stem cells. Cell Commun. Signal..

[28] Kim A, Ueda Y, Naka T, Enomoto T (2012). Therapeutic strategies in epithelial ovarian cancer. J. Exp. Clin. Cancer Res..

[29] West NR, Owens BMJ, Hegazy AN (2018). The oncostatin M-stromal cell axis in health and disease. Scand. J. Immunol..

[30] Nicola NA, Babon JJ (2015). Leukemia inhibitory factor (LIF). Cytokine Growth Factor Rev..

[31] Richards CD (2013). The enigmatic cytokine oncostatin m and roles in disease. ISRN Inflamm..

[32] Bryson BL, Junk DJ, Cipriano R, Jackson MW (2017). STAT3-mediated SMAD3 activation underlies Oncostatin M-induced senescence. Cell cycle.

[33] Guo L (2013). Stat3-coordinated Lin-28-let-7-HMGA2 and miR-200-ZEB1 circuits initiate and maintain oncostatin M-driven epithelial-mesenchymal transition. Oncogene.

[34] Godoy-Tundidor S (2005). Interleukin-6 and oncostatin M stimulation of proliferation of prostate cancer 22Rv1 cells through the signaling pathways of p38 mitogen-activated protein kinase and phosphatidylinositol 3-kinase. Prostate.

[35] West NR, Murray JI, Watson PH (2014). Oncostatin-M promotes phenotypic changes associated with mesenchymal and stem cell-like differentiation in breast cancer. Oncogene.

[36] Savarese TM (2002). Coexpression of oncostatin M and its receptors and evidence for STAT3 activation in human ovarian carcinomas. Cytokine.

[37] Chen JR, Zhao JT, Xie ZZ (2022). Integrin-mediated cancer progression as a specific target in clinical therapy. Biomed. Pharmacother..

[38] Scalici JM (2014). Inhibition of alpha4beta1 integrin increases ovarian cancer response to carboplatin. Gynecol. Oncol..

[39] Parashar D (2020). Peritoneal spread of ovarian cancer harbors therapeutic vulnerabilities regulated by FOXM1 and EGFR/ERBB2 Signaling. Cancer Res..

[40] Pradeep CR (2012). Modeling invasive breast cancer: growth factors propel progression of HER2-positive premalignant lesions. Oncogene.

[41] Moore, K. M. et al. Therapeutic targeting of integrin alphavbeta6 in breast cancer. *J. Natl Cancer Inst.*10.1093/jnci/dju169 (2014). 10.1093/jnci/dju169 PMC4151855 24974129

[42] Schachtrup C (2007). Fibrinogen inhibits neurite outgrowth via beta 3 integrin-mediated phosphorylation of the EGF receptor. Proc. Natl Acad. Sci. USA.

[43] Takada Y, Takada YK, Fujita M (2017). Crosstalk between insulin-like growth factor (IGF) receptor and integrins through direct integrin binding to IGF1. Cytokine Growth Factor Rev..

[44] Gao Y (2015). Up-regulation of CD44 in the development of metastasis, recurrence and drug resistance of ovarian cancer. Oncotarget.

[45] Zhang S (2008). Identification and characterization of ovarian cancer-initiating cells from primary human tumors. Cancer Res..

[46] Ganguly K (2021). Secretory mucin 5AC promotes neoplastic progression by augmenting KLF4-mediated pancreatic cancer cell stemness. Cancer Res..

[47] Jang TH (2022). MicroRNA-485-5p targets keratin 17 to regulate oral cancer stemness and chemoresistance via the integrin/FAK/Src/ERK/beta-catenin pathway. J. Biomed. Sci..

[48] Campbell PS (2018). AhR ligand aminoflavone suppresses alpha6-integrin-Src-Akt signaling to attenuate tamoxifen resistance in breast cancer cells. J. Cell Physiol..

[49] Dolinschek R (2021). Constitutive activation of integrin alphavbeta3 contributes to anoikis resistance of ovarian cancer cells. Mol. Oncol..

[50] Gasca J (2020). EDIL3 promotes epithelial-mesenchymal transition and paclitaxel resistance through its interaction with integrin alpha(V)beta(3) in cancer cells. Cell Death Discov..

[51] Uchihara T (2020). Extracellular vesicles from cancer-associated fibroblasts containing annexin A6 induces FAK-YAP activation by stabilizing beta1 integrin, enhancing drug resistance. Cancer Res..

[52] Fauci JM (2014). Monoclonal antibody-based immunotherapy of ovarian cancer: targeting ovarian cancer cells with the B7-H3-specific mAb 376.96. Gynecol. Oncol..

[53] Zhu X, Cai H, Zhao L, Ning L, Lang J (2017). CAR-T cell therapy in ovarian cancer: from the bench to the bedside. Oncotarget.

[54] Parashar, D. et al. Correction: Parashar et al. patient-derived ovarian cancer spheroids rely on pi3k-akt signaling addiction for cancer stemness and chemoresistance. Cancers 2022, 14, 958. *Cancers (Basel)* **14**, 10.3390/cancers14102443 (2022). 10.3390/cancers14102443 PMC9140135 35626175

[55] Pijuan J (2019). In vitro cell migration, invasion, and adhesion assays: from cell imaging to data analysis. Front. Cell Dev. Biol..

[56] Arora, M. et al. Involvement of DPP3 in modulating oncological features and oxidative stress response in esophageal squamous cell carcinoma. *Biosci. Rep.*10.1042/BSR20222472 (2023). 10.1042/BSR20222472 PMC10500228 37531267

[57] George J (2021). RNA-binding protein FXR1 drives cMYC translation by recruiting eIF4F complex to the translation start site. Cell Rep..

[58] Gupta P (2022). Tumor derived extracellular vesicles drive T Cell exhaustion in tumor microenvironment through sphingosine mediated signaling and impacting immunotherapy outcomes in ovarian cancer. Adv. Sci..

[59] Goedhart J, Luijsterburg MS (2020). VolcaNoseR is a web app for creating, exploring, labeling and sharing volcano plots. Sci. Rep..

