# Supplementary material for: Blocking Oncostatin M receptor abrogates STAT3 mediated integrin signaling and overcomes chemoresistance in ovarian cancer
Source: NPJ Precis Oncol. 2024 Jun 5;8:127. doi: 10.1038/s41698-024-00593-y (PMC11153533; doi:10.1038/s41698-024-00593-y)
Supplement: Supplementary file 1 — Combined supplementary materials [file 41698_2024_593_MOESM1_ESM.pdf]

Combined Supplementary Materials

Supplementary Fig 1

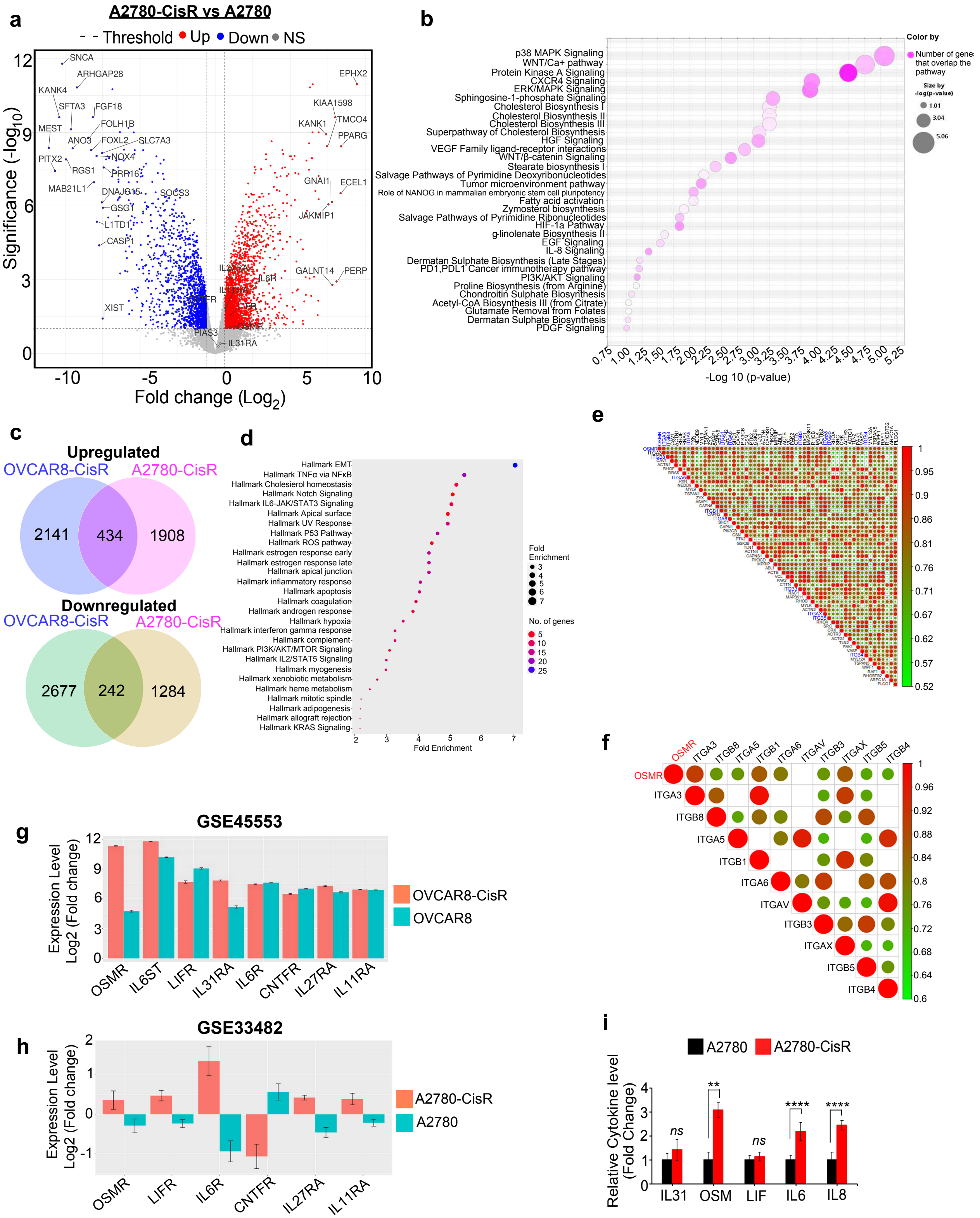

Supplementary Fig. 1 Oncostatin M (OSM) receptor is upregulated in chemoresistant ovarian cancer and is associated with integrin signaling pathways. **a** Volcano plot shows genes which are differentially expressed in A2780-CisR (cisplatin resistant) (n=6) compared with A2780 sensitive (parental) cell lines (n=6) in a publicly available microarray dataset (GSE33482). Genes with log2 (fold change) threshold set at +/-0.58 with a pValue <0.05 and FDR <0.1 were selected. Highly differentially expressed genes and genes associated with oncostatin receptor pathways were labeled. **b,** IPA analysis of canonical pathways identified using the upregulated genes that are > 1.5-fold change in A2780-CisR cell lines from (A). **c** Venn diagram shows commonly upregulated (FC: ≥ 1.5) and downregulated (FC ≤ 0.67) genes with a pValue <0.05 and FDR <0.1 in GSE45553 and GSE33482 microarray datasets. **d** Hallmarks of cancer associated pathways identified from the commonly upregulated 434 genes from C using ShinyGO gene enrichment analysis program. **e, f** Correlation dot plot shows the Spearman's correlation of OSMR with (e) Integrin family genes and (f) with integrins α and β genes exhibiting pValue ≤ 0.05 in GSE45553 OVCAR8-CisR microarray dataset. **g, h** Expression levels of IL6 receptor family genes in GSE45553 and GSE33482 microarray datasets respectively. **i** ELISA was performed to determine the levels of the ligands of IL6 family receptors such as OSM, LIF, IL31, IL6 and IL8 in culture supernatant of A2780-CisR *vs.* A2780 sensitive cell lines. Student's t test was performed to determine significance between different groups. Error bars represent mean ± SEM**.** ****P≤0.0001, **P≤0.01, ns: non-significant.

Supplementary Figure 2

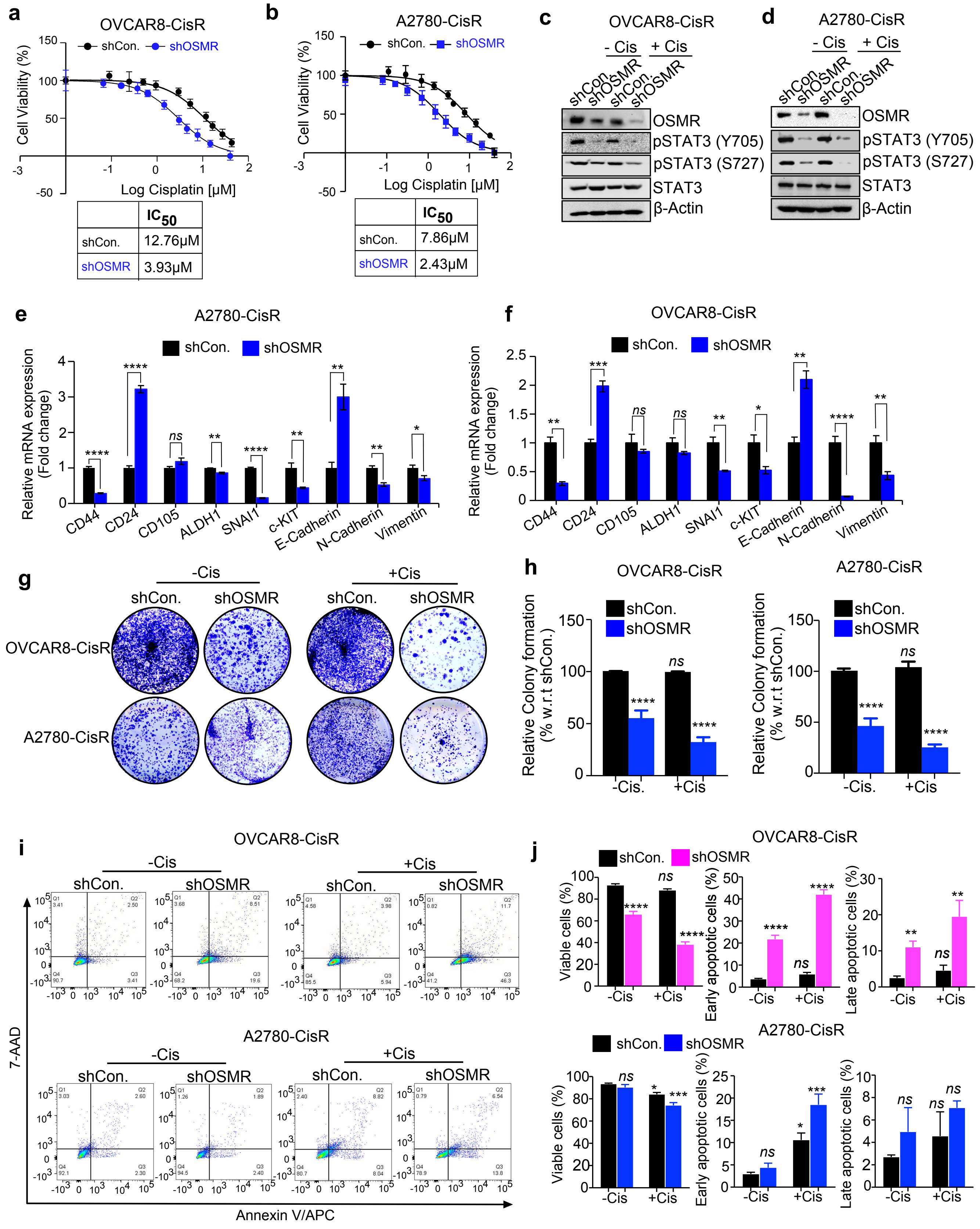

Supplementary Fig. 2 OSMR knockdown abrogates chemoresistance by enhancing apoptosis

a-b OVCAR8-CisR and A2780-CisR cell lines stably knockdown with shOSMR were treated with varying concentrations of cisplatin as indicated for 48h and cell viability or sensitivity towards cisplatin was assessed using CCK8 assay. IC_{50} of cisplatin for each cell line are shown in the box. **c-d** OVCAR8-CisR and A2780-CisR cell lines stably knockdown with shOSMR were treated without and with cisplatin and immunoblotting with indicated antibodies were performed. **e, f** RNA was isolated from OVCAR8-CisR and A2780-CisR cell lines stably knockdown for OSMR using shOSMR and qPCR was performed to determine the mRNA expression of the indicated genes. B-Actin was used as the internal standard. **g** OVCAR8-CisR and A2780-CisR cell lines stably knockdown OSMR with shOSMR and the respective controls were treated without or with cisplatin and grown for 15 days. The colonies formed were stained using 0.5 % crystal violet, and imaged. **h** Crystal violet-stained colonies were dissolved in 10% acetic acid and absorbance was measured at 560 nm and quantitated. **i** OVCAR8-CisR and A2780-CisR cell lines stably knockdown of OSMR with shOSMR were treated without and with cisplatin for 16h and the level of cellular apoptosis was determined using Annexin V-APC/7-AAD staining followed by flow cytometry. **j** Average percentage of viable cells (Q4), early apoptotic cells (Q3) and late apoptotic cells (Q2) were quantitated from three separate experiments. Student's t test was performed to determine significance between two groups. Error bars represent mean ± SEM**.** ****P≤0.0001, ***P≤0.001, **P≤0.01, *P≤0.05.

Supplementary Figure 3

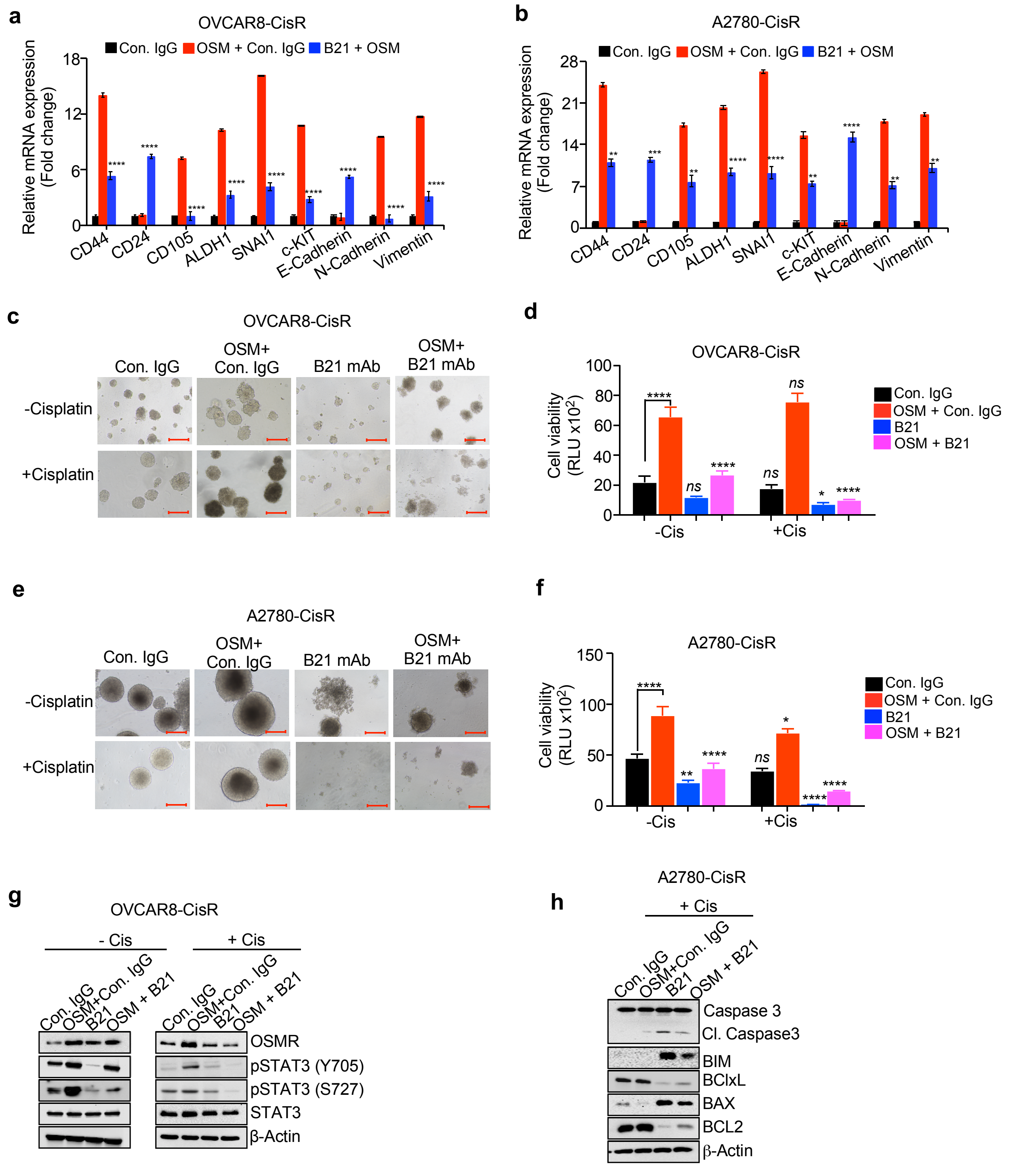

Supplementary Fig. 3 Anti-OSMR antibody enhances sensitivity towards cisplatin.

a-b RNA was isolated from OVCAR8-CisR and A2780-CisR cell lines treated with B21 in combination without and with cisplatin in the presence and absence of rhOSM for 48h. Then, total RNA was isolated, and qPCR was performed to determine the mRNA expression of the indicated genes. β-Actin was used as an internal standard **c, e** 3-D spheroid formation was performed in A2780-CisR and OVCAR8-CisR cell lines treated with B21 antibody (10 μg/mL each) with and without cisplatin and in the presence and absence of rhOSM (100 ng/mL) in clonacell media: complete DMEM (1:1) and seeded on ultra-low attachment plate for 15 days to assess the spheroid forming ability and imaged. Scale bar, 500 μM. Cell viability of 3D spheroids were determined using 3D-Cell Titer-Glow reagent. **d, f** The histograms show the luminescence intensity measured using 3D-viability assay kit which corresponds to cell viability of spheroids. **g** Western blot analysis showing the expression of indicated proteins in OVCAR8-CisR resistant cell lines that were treated with B21 anti-OSMR antibody or control IgG (10 µg/mL) for 60 min in combination with cisplatin followed by stimulation with rhOSM (100 ng/mL). **g** Western blot analysis showing the expression of indicated pro-apoptotic and anti-apoptotic proteins in A2780-CisR resistant cell lines that were treated with B21 anti-OSMR antibodies (10 µg/mL) in the absence or presence of rhOSM (100 ng/mL) followed by treatment with or without cisplatin for 24 h. Student's t test was performed to determine significance in 'a-b'. One-way ANOVA followed by Dunnett's multiple comparison test were performed to determine significance in 'd-f'. Error bars represent mean ± SEM**.** ****$P \leq 0.0001$, ***$P \leq 0.001$, **$P \leq 0.01$, *$P \leq 0.05$.

Supplementary Fig 4

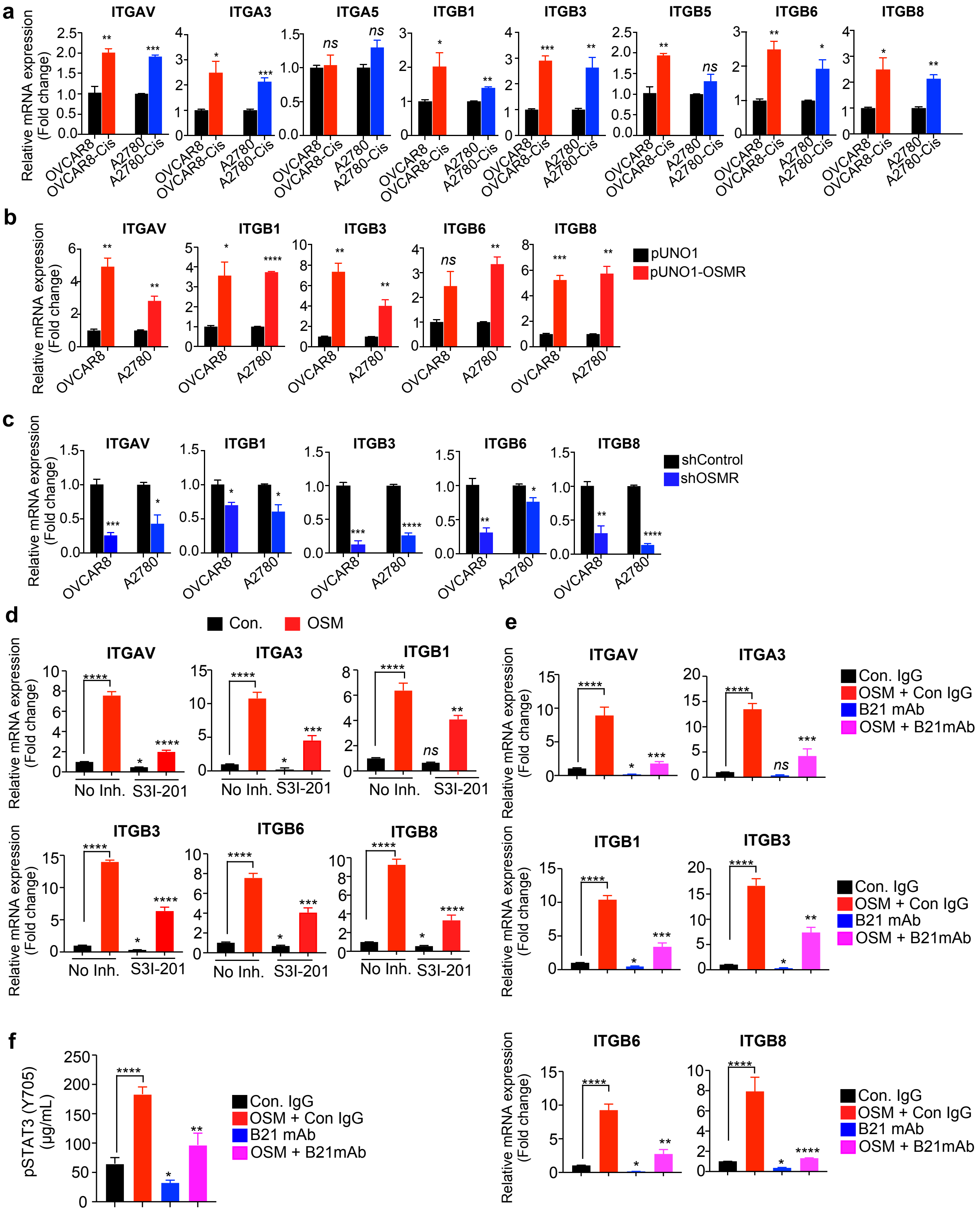

Supplementary Fig. 4 OSMR relies on integrin signaling to promote chemoresistance.

a RNA was isolated from OVCAR8, OVCAR8-CisR, A2780, and A2780-CisR cell lines and qPCR was performed to determine the mRNA expression of the indicated genes. The histograms show relative mRNA expression w.r.t respective sensitive cell lines. β-Actin was used as internal control. **b** OVCAR8 and A2780 cell lines were stably overexpressed with pUNO1-OSMR plasmid and qPCR was performed to determine mRNA expression of the indicated genes. The histograms show relative mRNA expression w.r.t respective empty vector control (pUNO1). **c** OSMR was stably knockdown in OVCAR8-CisR and A2780-CisR cell lines using shRNA and qPCR was performed to determine mRNA expression of the indicated genes. The histograms show relative mRNA expression w.r.t respective shRNA control. **d** OVCAR8 cell lines were treated without and with S3I-201 inhibitor for 30min followed by stimulation in the absence and presence of rhOSM (100 ng/mL) for 48 h. RNA was isolated, and qPCR was performed to determine mRNA expression of the indicated genes. The histograms show relative mRNA expression w.r.t no inhibitor control *vs.* inhibitor and OSM versus OSM+S3I-201 inhibitor. **e** OVCAR8 cell lines were treated with B21 anti-OSMR antibody or control IgG alone or in combination with rhOSM (100 ng/mL) for 30 min and pSTAT3 (Y705) levels were determined by ELISA. Student's t-test was performed to determine significance in 'a-c'. One-way ANOVA followed by Dunnett's multiple comparison test were performed to determine significance in 'd-f'. Error bars represent mean ± SEM**.** ****P≤0.0001, ***P≤0.001, **P≤0.01, *P≤0.05, ns: non-significant.

Supplementary Fig 5

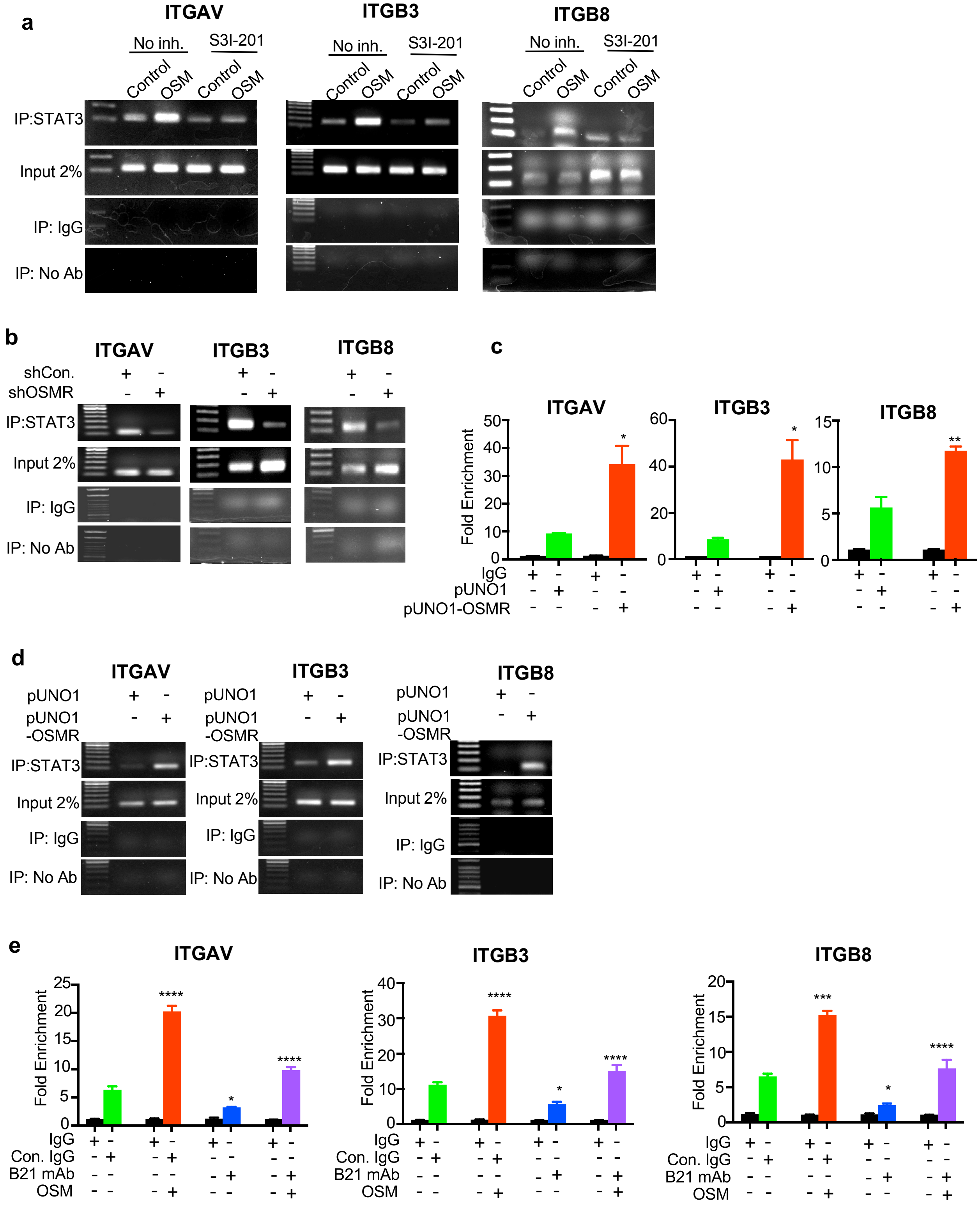

Supplementary Fig. 5 OSMR regulates the expression of integrins transcriptionally via STAT3.

a OVCAR8 cells were treated without and with S3I-201 inhibitor for 30 min followed by stimulation of rhOSM (100 ng/mL) for 48 h and were cross-linked, harvested and subjected to immunoprecipitation with STAT3 antibody. DNA was isolated from STAT3 bound chromatin elutes and PCR was performed. The representative images show the amplified STAT3-bound integrin DNA run on 1 % agarose gel. The ladder used is 100 bp. **b** The representative images show the amplified STAT3-bound integrin DNA from 'Fig 4F, and 4G were run on 1 % agarose gel. Blank refers to 'no DNA' negative control and IgG is used as negative control to calculate relative fold enrichment. **c** OVCAR8 cells stably overexpress OSMR, and their control cells were cross-linked, harvested and subjected to immunoprecipitation with STAT3 antibody. DNA was isolated from STAT3 bound chromatin elutes and qPCR was performed. **d** Representative images show the amplified STAT3-bound integrin DNA from 'C' were run on 1 % agarose gel. **e** OVCAR8 cells were treated with control IgG (Isotype control) or B21 alone and in combination with rhOSM stimulation for 48 h and were cross-linked, harvested and subjected to immunoprecipitation with STAT3 antibody. DNA was isolated from STAT3 bound chromatin elutes and qPCR was performed. The histograms show fold enrichment of the indicated integrin promoters bound with STAT3 w.r.t IgG. P-values were determined w.r.t Control IgG *vs.* B21 and OSM + Con. IgG *vs* OSM+B21. Student's t test was performed to determine significance in 'c'. One-way ANOVA followed by Dunnett's multiple comparison test were performed to determine significance in 'e'. Error bars represent mean ± SEM. ****P≤0.0001, ***P≤0.001, **P≤0.01, *P≤0.05.

Supplementary Fig 6

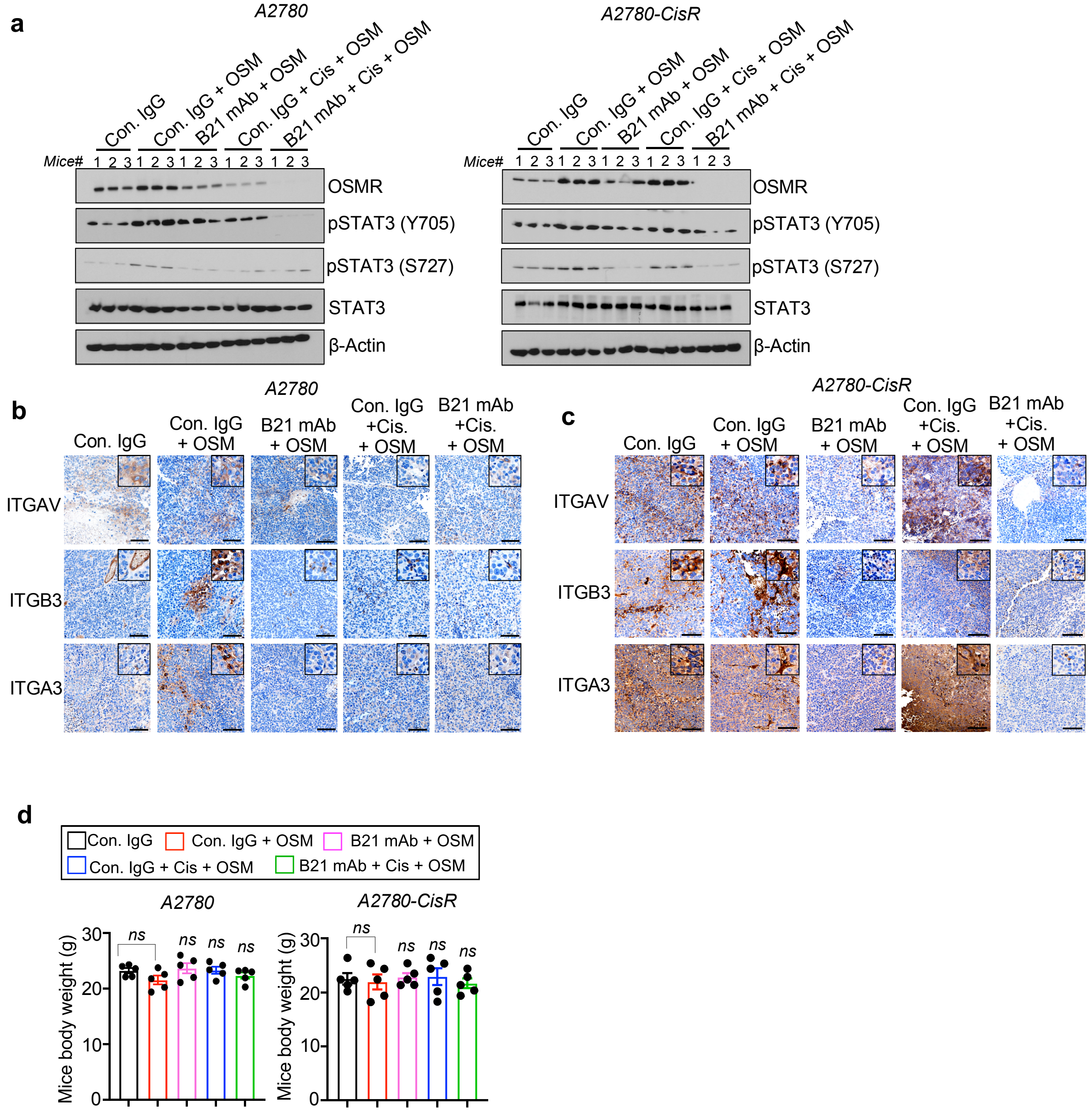

Supplementary Fig 6 Anti-OSMR antibodies promote sensitization towards cisplatin therapy in cisplatin resistant ovarian cancer cells *in vivo*.

a Athymic nude mice were injected intraperitoneally (i.p) with A2780-Luc+ (n=5) and A2780-CisR-Luc+ (n=5) as indicated in Fig 5a. Respective groups were treated with isotype control IgG or B21 mAb in the presence and absence of Cisplatin and stimulated with and without rhOSM. Tumor tissues from three mice per group were homogenized, lysed and protein lysates were prepared and immunoblotted. **b, c** The tumor tissues from 'a' were fixed, paraffin embedded and sectioned and immunohistochemistry was performed using the antibodies indicated. The scale bar indicates 100 μm. **d** Body weight of mice bearing A2780 and A2780-CisR treatment groups described in Fig 5a were recorded before the termination of experiment on day 35. One-way ANOVA followed by Dunnett's multiple comparison test were performed to determine significance in 'd'. Error bars represent mean ± SEM**.** ****P≤0.0001, ***P≤0.001, **P≤0.01, *P≤0.05, ns: non-significant.

Supplementary Fig 7: Uncropped images of Western blots included in Figure-1, Figure-2, Figure-3, Figure-4, Supplementary Figure-2, Supplementary Figure-3, and Supplementary Figure-6 with labels of molecular weight markers:

Figure 1

F

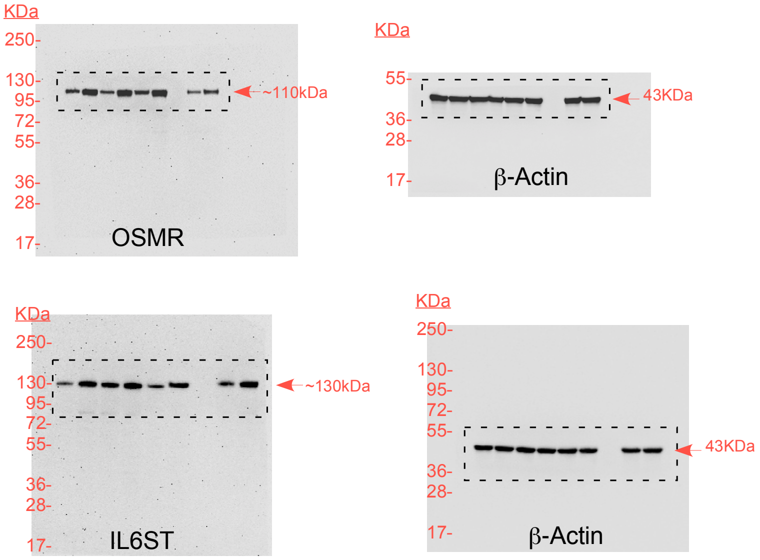

G

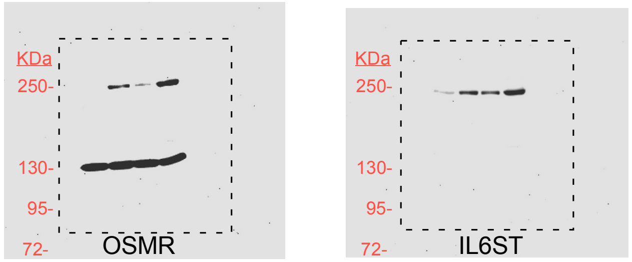

H

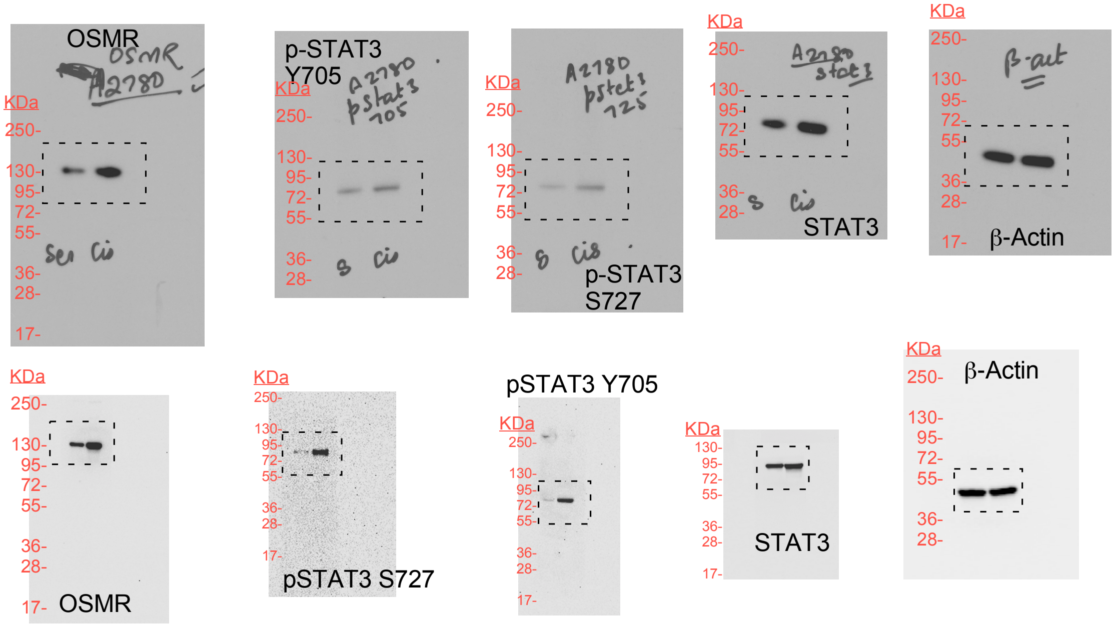

Figure 2

c

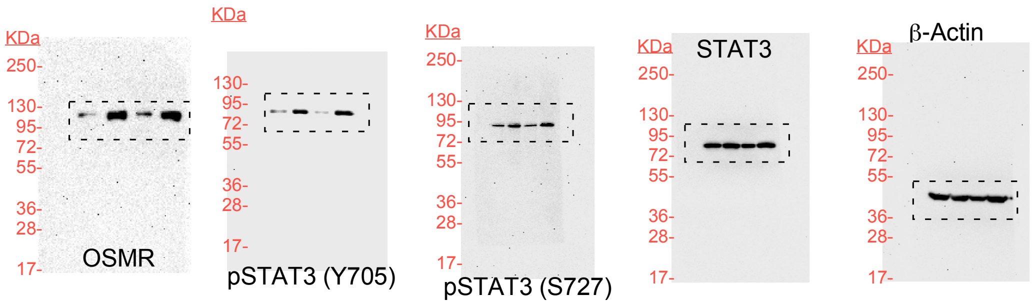

d

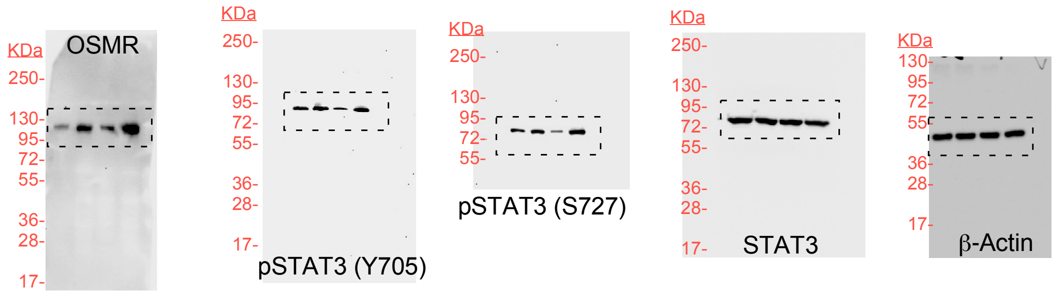

Fig 3G

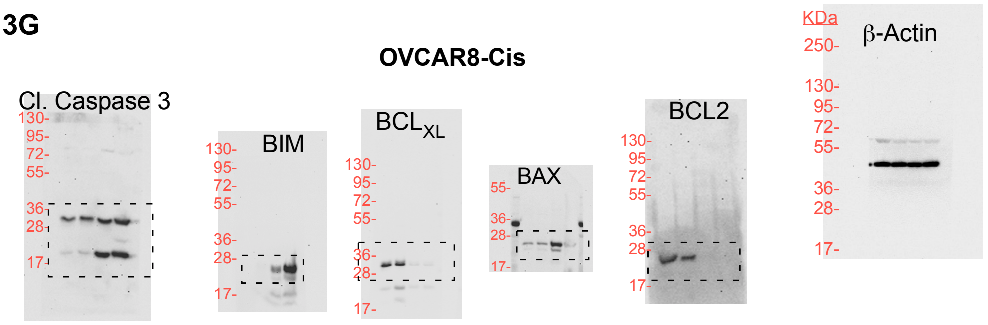

Figure 4

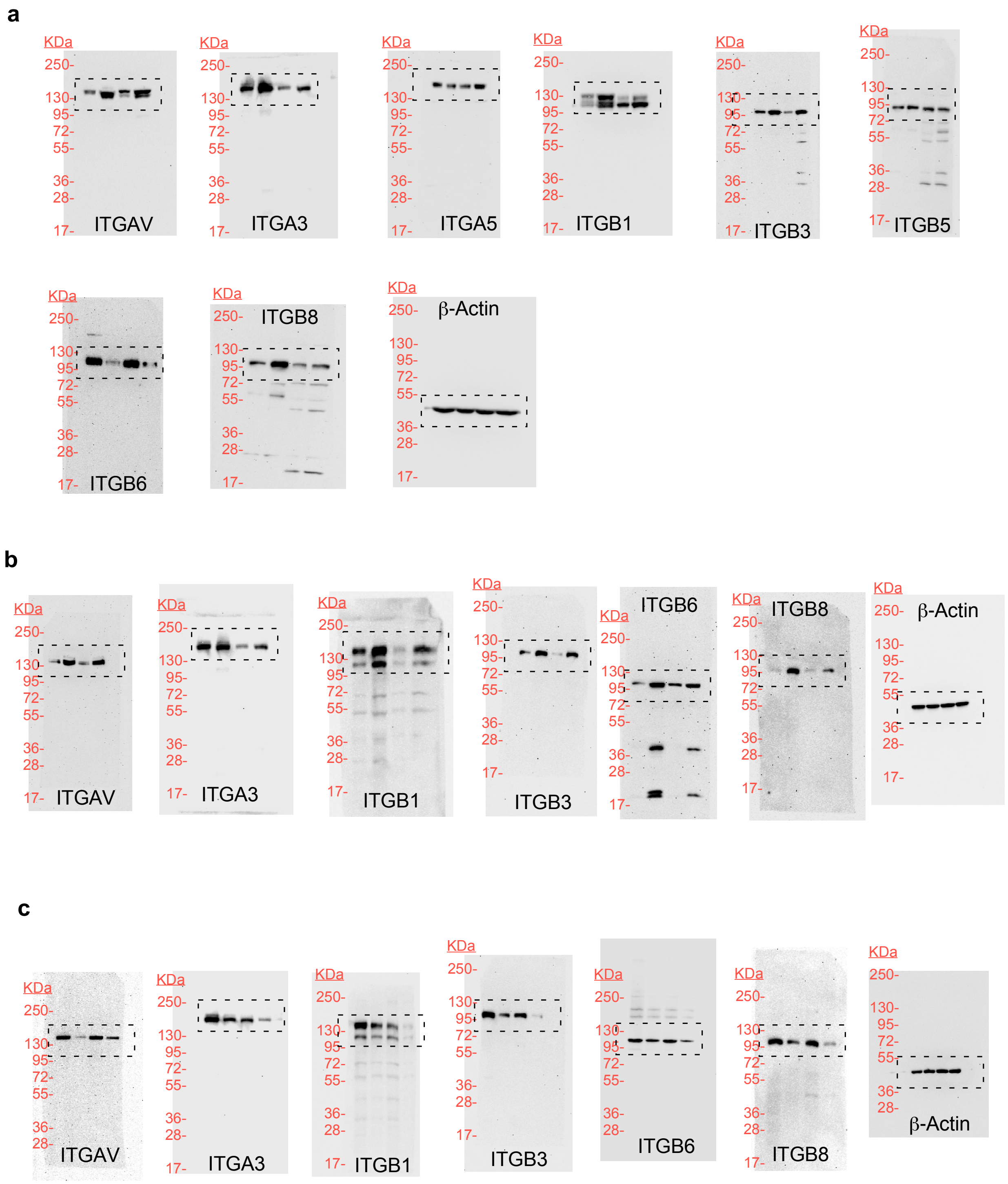
a
KDa
250-
130-
95-
72-
55-
36-
28-
17-
ITGAV
ITGA3
ITGA5
ITGB1
ITGB3
ITGB5
ITGB6
ITGB8
β-Actin
b
ITGAV
ITGA3
ITGB1
ITGB3
ITGB6
ITGB8
β-Actin
c
ITGAV
ITGA3
ITGB1
ITGB3
ITGB6
ITGB8
β-Actin

Supplementary Fig 2

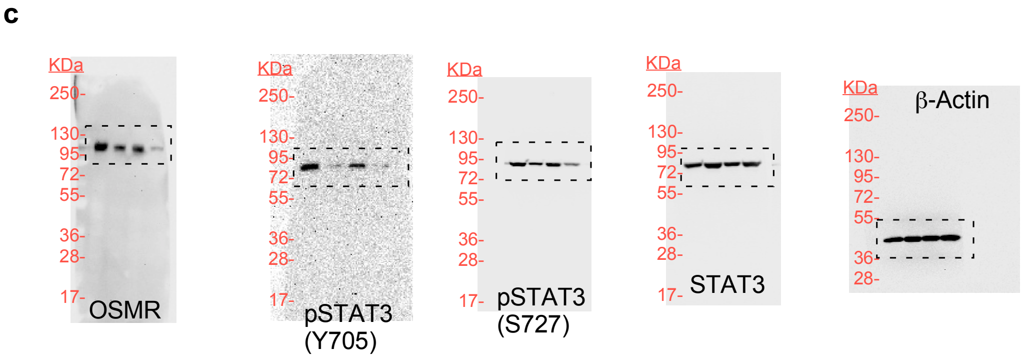

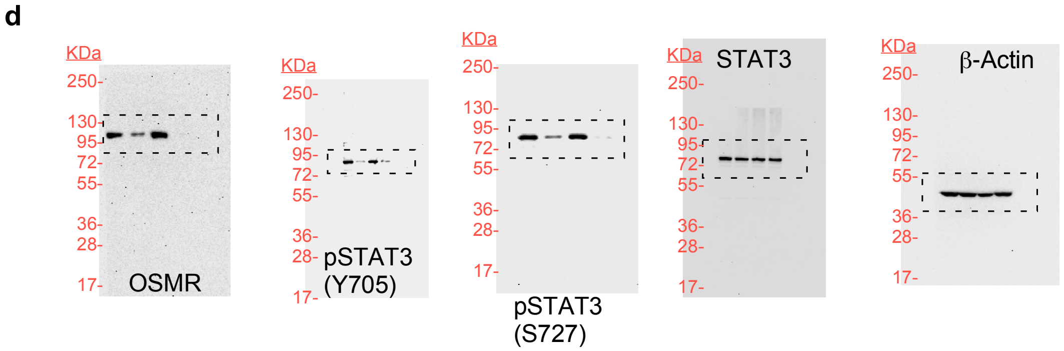

Supplementary Fig 3

g

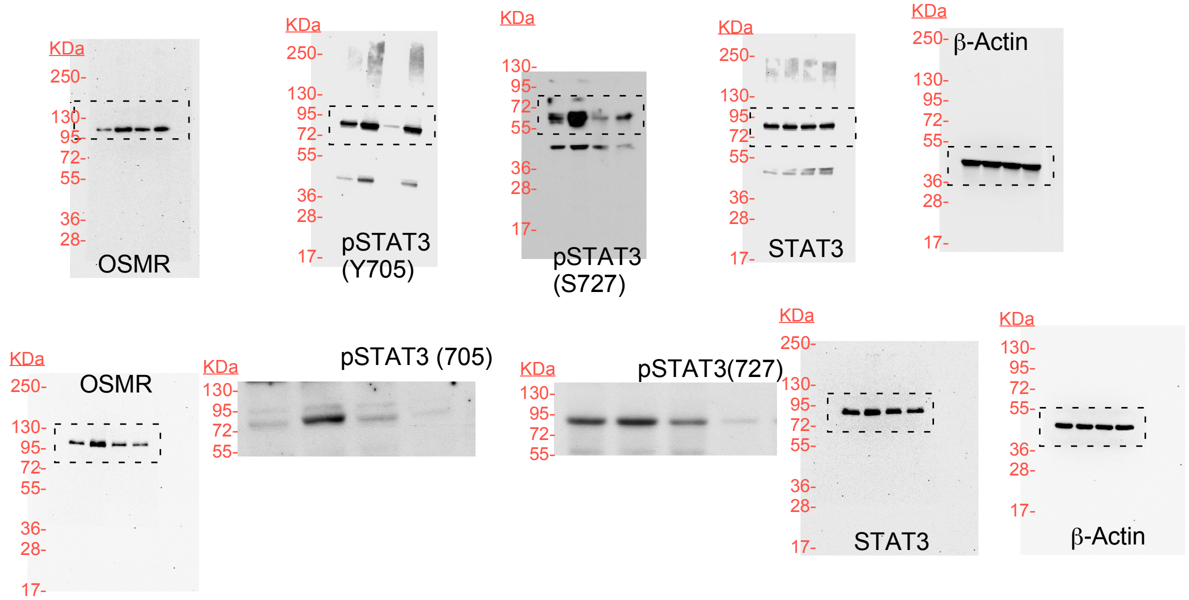

h

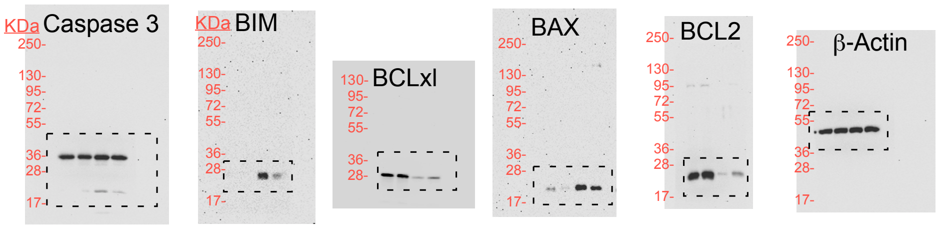

Supplementary Figure 6

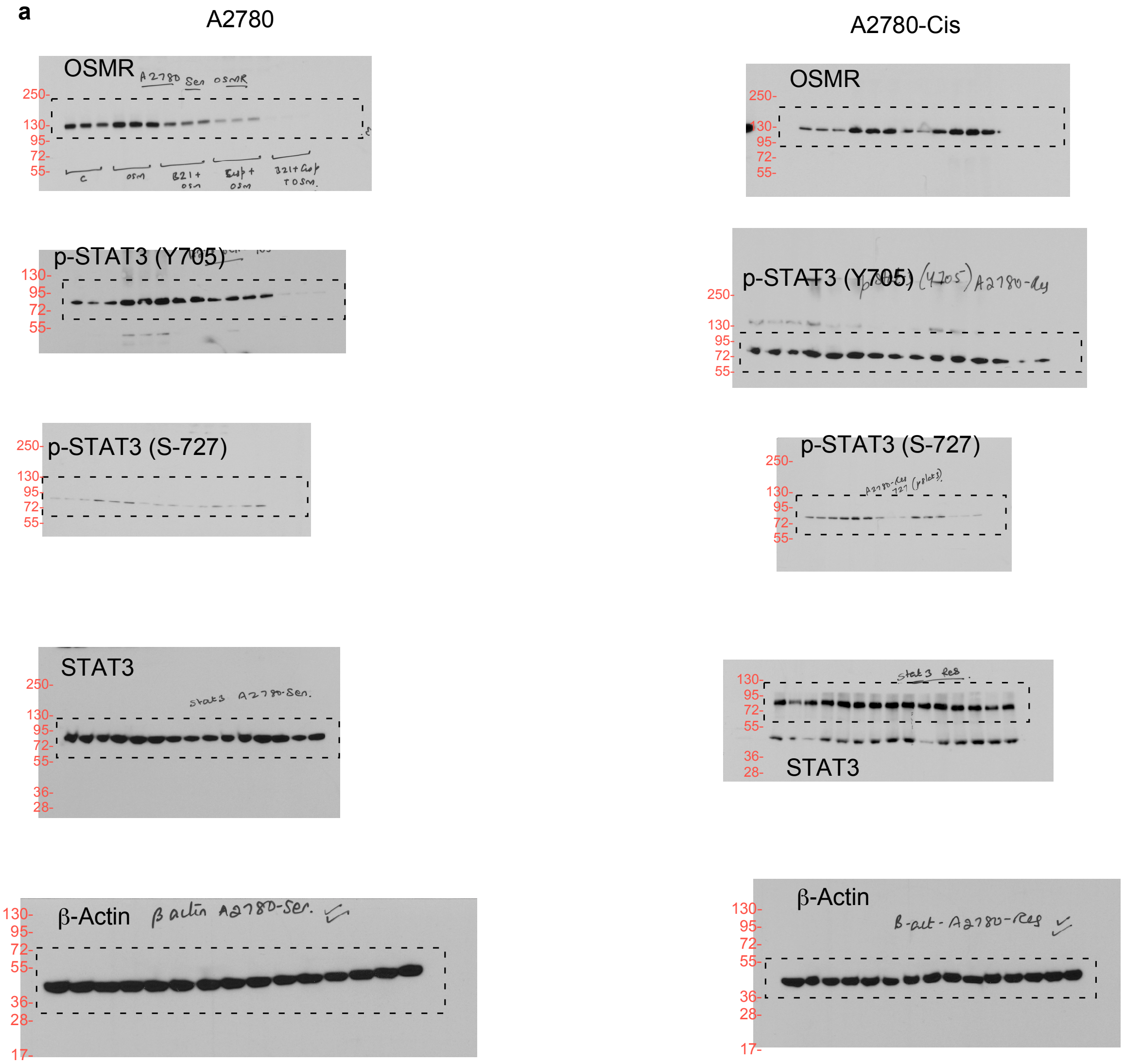

Supplementary Table S1. List of Antibodies and Reagents used

**Antibodies**			
**Reagent**	**Source**	**Identifier**	**Dilution**
Anti-OSMR	Proteintech	Cat#: 10982-1-AP	1:1,000
Anti- STAT3 (phospho Y705)	Cell Signaling Technology	Cat#: 9145	1:1,000
Anti- STAT3 (phospho S727)	Cell Signaling Technology	Cat#: 9134	1:1,000
Anti- STAT3	Cell Signaling Technology	Cat#: 9139	1:1,000
Anti-IL6ST/gp130	Santa Cruz Biotechnologies	Cat#: sc-376280	1:500
Anti- β-Actin	Santa Cruz Biotechnologies	Cat#: sc-47778	1: 1,000
Anti-BCL2	Cell Signaling Technology	Cat#: 15071	1:1,000

Anti- BCLxL	Cell Signaling Technology	Cat#: 2764	1: 1,000
Anti- Cleaved caspase 3	Cell Signaling Technology	Cat#: 9661	1:700
Anti-caspase 3	Cell Signaling Technology	Cat#: 14220	1: 1,000
Anti-BIM	Cell Signaling Technology	Cat#: 2933	1: 1,000
Anti-BAX	Cell Signaling Technology	Cat#: 2772	1:1000
Anti-ITGAV	Cell Signaling Technology	Cat#: 60896	1:1000
Anti-ITGA3	Proteintech	Cat#: 21992	1:1200
Anti-ITGA5	Cell Signaling Technology	Cat#: 98204	1:1000

Anti-ITGB1	Cell Signaling Technology	Cat#: 34971	1:1000
Anti-ITGB3	Cell Signaling Technology	Cat#: 4702	1:1000
Anti-ITGB5	Cell Signaling Technology	Cat#: 4708	1:1000
Anti-ITGB6	Cell Signaling Technology	Cat#: 95153	1:1000
Anti-ITGB8	Thermo Fisher Scientific	Cat#: PA5-100843	1:1000
Anti-Ki67	Cell Signaling Technology	Cat#: 9449	1:1000
Anti-rabbit IgG	Cell Signaling Technology	Cat#: 7074	1:3000
Anti-mouse IgG	Cell Signaling Technology	Cat#: 7076	1:3000

**Chemicals and assay kits**			
**Reagent**	**Source**	**Identifier**	**Use**
Cisplatin	Selleckchem	Cat#: S1166	In vitro & in vivo treatments
Crytotanshinone	Selleckchem	Cat#: S2285	In vitro treatments
S3I-201	Selleckchem	Cat#: S1155	In vitro treatments
RGD peptide inhibitor	Selleckchem	Cat#: S4513	In vitro treatments
Fibronectin	Sigma Aldrich	Cat#: F0895	In vitro treatments
Recombinant Human OSM	Sino Biologicals	Cat#: 10452-HNAH	In vitro & in vivo treatments
Cell Counting Kit-8 kit	Dojindo Molecular Technologies, Rockville, MD	Cat#: CK04	Cell viability
ClonaCell™-TCS Medium	Stemcell Technologies Seattle, WA	Cat#: 03814	Colonogenic assay

Luminex Multiplex ELISA	R&D Systems	Cat#: LXSAHM-05	Multiplex cytokine ELISA
BCA kit	Thermo Fisher Scientific	Cat#: J63283-QA	Protein estimation
BS3	Thermo Fisher Scientific	Cat#: 21580	Heterodimerization
Human IL-6 Signaling Pathway RT2 Profiler™	Qiagen, Frederick, MD., USA	Cat#: 330231, PAHS-160ZA	qPCR Array
SimpleChIP® Enzymatic Chromatin IP Kit	Cell Signaling Technology	Cat#: 9003	Chromatin IP
Annexin V-FITC-PI	Biolegend	Cat#: 640914	Flow Cytometry

Annexin V-APC-7-AAD	Biolegend	Cat#: 640930	Flow Cytometry
ImmPRESS HRP Horse Anti-Rabbit IgG PLUS Polymer Kit, Peroxidase	Vector Laboratories	Cat#: MP-7801-15	Immunohistochemistry
ImmPRESS HRP Horse Anti-Mouse IgG PLUS Polymer Kit, Peroxidase	Vector Laboratories	Cat#: MP-7802-15	Immunohistochemistry
IHC-Tek Epitope Retrieval Solution	IHC-World, MD	Cat#: IW-1100	Immunohistochemistry

Software			
**Software**	**Version**	**Source**	**Purpose**
VolcaNoseR		Goedhart, J., Luijsterburg, M.S. VolcaNoseR is a web app for creating, exploring, labeling and sharing volcano plots. Sci Rep 10, 20560 (2020). https://huygens.science.uva.nl/VolcaNoseR/	Volcano plot maker
Graph Pad	GraphPad Prism version 9.3.0	-	Statistical analysis
Venn Diagram	Venny 2.1.0	Oliveros, J.C. (2007-2015) Venny. An interactive tool for comparing lists with Venn's diagrams. https://bioinfogp.cnb.csic.es/tools/venny/index.html	Venn diagram maker
ShinyGO	ShinyGO 0.76.3	Steven Xijin Ge, Dongmin Jung, Runan Yao, ShinyGO: a graphical gene-set enrichment tool for animals and plants, Bioinformatics, Volume 36, Issue 8, 15 April 2020, Pages 2628–2629,	GO analysis

		http://ge-lab.org/go/	

Supplementary Table S2. List of qRT-PCR primer sequences

Name	Sequence (5’-3’)
CD44 Forward	CTG CCG CTT TGC AGG TGT A
CD44 Reverse	CAT TGT GGG CAA GGT GCT ATT
CD24 Forward	CTC CTA CCC ACG CAG ATT TAT TC
CF24 Reverse	AGA GTG AGA CCA CGA AGA GAC
CD105 Forward	TGC ACT TGG CCT ACA ATT CCA
CD105 Reverse	AGC TGC CCA CTC AAG GAT CT
ALDH1 Forward	CCA TAA CAA TCT CCT CTG CTC TG
ALDH1 Reverse	CTC TCC CAG TTC TCT TCC ATT TC
SNAI1 Forward	CCT TCG TCC TTC TCC TCT ACT T
SNAI1 Reverse	TTC GAG CCT GGA GAT CCT T
KIT Forward	CGT TCT GCT CCT ACT GCT TCG
KIT Reverse	CCC ACG CGG ACT ATT AAG TCT
E-Cadherin Forward	GCT GGA CCG AGA GAG TTT CC
E-Cadherin Reverse	CAA AAT CCA AGC CCG TGG TG
N-Cadherin Forward	CCT GCT TAT CCT TGT GCT GAT G
N-Cadherin Reverse	TGG TCT TCT TCT CCT CCA CCT
Vimentin Forward	GCA GGA GGC AGA AGA ATG GT
Vimentin Reverse	CCA CTT CAC AGG TGA GGG AC
B-Actin Forward	CAT GTA CGT TGC TAT CCA GGC
B-Actin Reverse	CTC CTT AAT GTC ACG CAC GAT
ITGAV Forward	GCT GTC GGA GAT TTC AAT GGT

ITGAV Reverse	TCT GCT CGC CAG TAA AAT TGT
ITGA5 Forward	GGC TTC AAC TTA GAC GCG GAG
ITGA5 Reverse	TGG CTG GTA TTA GCC TTG GGT
ITGB1 Forward	CAA GAG AGC TGA AGA CTA TCC CA
ITGB1 Reverse	TGA AGT CCG AAG TAA TCC TCC T
ITGB3 Forward	AGT AAC CTG CGG ATT GGC TTC
ITGB3 Reverse	GTC ACC TGG TCA GTT AGC GT
ITGB8 Forward	GTG AAA GTC ATA TCG GAT GGC G
ITGB8 Reverse	GCT ATC AAG AGC GAG ATG AGA CG
ITGB6 Forward	TCC ATC TGG AGT TGG CGA AAG
ITGB6 Reverse	TCT GTC TGC CTA CAC TGA GAG
ITGB5 Forward	GGA AGT TCG GAA ACA GAG GGT
ITGB5 Reverse	CTT TCG CCA GCC AAT CTT CTC
IL6ST Forward	GCA ACA CAC AAG TTT GCT GAT T
IL6ST Reverse	CCT TCC CAA GGG CAT TCT CTG
LIFR Forward	TGG AAC GAC AGG GGT TCA GT
LIFR Reverse	GAG TTG TGT TGT GGG TCA CTA A
IL11RA Forward	CTG GGC TAG GGC ATG AAC TG
IL11RA Reverse	CTG GGA CTC CAA GTG CAA GA
IL27RA Forward	CCC CGT CTT CGT GAA CCT AGA
IL27RA Reverse	ACA TCT TCG GTA GTG GAA CTG G
IL6R Forward	CCA TGC AGG CAC TTA CTA CT
IL6R Reverse	GGC AGT GGT ACT GAA GAA GAA

IL31RA Forward	TGT TCC ACC CCC AGT GAC AA
IL31RA Reverse	AGT CCC ATT CTT TTC CCC TCC T
CNTFR Forward	CAG CAC ACA CCA TCA CAG A
CNTFR Reverse	GTC ACT CCA TGT CCC AAT CTC
ITGA3 Forward	TGT GGC TTG GAG TGA CTG TG
ITGA3 Reverse	TCA TTG CCT CGC ACG TAG C
OSMR Forward	AAT GTC AGT GAA GGC ATG AAA GG
OSMR Reverse	GAA GGT TGT TTA GAC CAC CCC

Supplementary Table S3. List of qRT-PCR promoter primer sequences

Name	Sequence (5’-3’)
ITGAV Forward	CTG CGA ATC CTT TCT TTG ACT AC
ITGAV Reverse	CTC CAT TCC ACA AAC ACT GAA C
ITGB3 Forward	CAT GGA CCT ATC ACT GCT TAC G
ITGB3 Reverse	CAT CTG CTG AAT GCC TTC TCT
ITGB8 Forward	CCT CGA CTT AGC GTG GTA ATG
ITGB8 Reverse	CTG CTT GCA GCT CAG AAA TTT AG
