# Supplementary material for: Blocking Oncostatin M receptor abrogates STAT3 mediated integrin signaling and overcomes chemoresistance in ovarian cancer
Source: NPJ Precis Oncol. 2024 Jun 5;8:127. doi: 10.1038/s41698-024-00593-y (PMC11153533; doi:10.1038/s41698-024-00593-y)
Supplement: Supplementary file 2 — REPORTING SUMMARY [file 41698_2024_593_MOESM2_ESM.pdf]

nature portfolio

Corresponding author(s): Pradeep Chaluvally-Raghavan, Zhiqiang An

Last updated by author(s): Mar 21, 2024

Reporting Summary

Nature Portfolio wishes to improve the reproducibility of the work that we publish. This form provides structure for consistency and transparency in reporting. For further information on Nature Portfolio policies, see our Editorial Policies and the Editorial Policy Checklist.

Statistics

For all statistical analyses, confirm that the following items are present in the figure legend, table legend, main text, or Methods section.

n/a	Confirmed	
☐	☒	The exact sample size (*n*) for each experimental group/condition, given as a discrete number and unit of measurement
☐	☒	A statement on whether measurements were taken from distinct samples or whether the same sample was measured repeatedly
☐	☒	The statistical test(s) used AND whether they are one- or two-sided<br>*Only common tests should be described solely by name; describe more complex techniques in the Methods section.*
☒	☐	A description of all covariates tested
☐	☒	A description of any assumptions or corrections, such as tests of normality and adjustment for multiple comparisons
☐	☒	A full description of the statistical parameters including central tendency (e.g. means) or other basic estimates (e.g. regression coefficient) AND variation (e.g. standard deviation) or associated estimates of uncertainty (e.g. confidence intervals)
☐	☒	For null hypothesis testing, the test statistic (e.g. *F*, *t*, *r*) with confidence intervals, effect sizes, degrees of freedom and *P* value noted<br>*Give P values as exact values whenever suitable.*
☒	☐	For Bayesian analysis, information on the choice of priors and Markov chain Monte Carlo settings
☒	☐	For hierarchical and complex designs, identification of the appropriate level for tests and full reporting of outcomes
☐	☒	Estimates of effect sizes (e.g. Cohen's *d*, Pearson's *r*), indicating how they were calculated

Our web collection on statistics for biologists contains articles on many of the points above.

Software and code

Policy information about availability of computer code

Data collection	Gene expression microarray profiling data, GSE45553 and GSE33482, were obtained online from Gene Expression Omnibus (http://www.ncbi.nlm.nih.gov/geo), Spearman's correlation analysis was performed to analyze the correlation between OSMR and integrin family genes from TCGA-OV dataset consisting of 379 Ovarian Serous Cystadenocarcinoma patient cohort was downloaded from GDC data portal (https://portal.gdc.cancer.gov) via Bioconductor package TCGA and gene expression profiles were processed via HTSeq pipeline.
Data analysis	Differentially expressed genes were identified by DESeq2 using R software, Pathway analysis of significantly differentially expressed genes was conducted using Ingenuity Pathway Analysis (IPA) to identify the canonical pathways, Volcano plot was prepared using VolcaNoseR, Spearman's correlation analysis was performed to analyze the correlation between OSMR and integrin family genes from TCGA-OV dataset consisting of 379 Ovarian Serous Cystadenocarcinoma patient cohort was downloaded from GDC data portal (https://portal.gdc.cancer.gov) via Bioconductor package TCGA and gene expression profiles were processed via HTSeq pipeline, Correlation plots of Integrin signaling pathway genes and integrin genes with OSMR from the microarray dataset (GSE45553) were plotted using R software, Venn diagram was prepared using Venny 2.1.0, ShinyGO 0.76.3 was used for GeneOntology analysis, GraphPad Prism version 9.3.0 was used to analyze cell viability assay data and both GraphPad and Microsoft Excel were used to graphically display the analyzed data.

For manuscripts utilizing custom algorithms or software that are central to the research but not yet described in published literature, software must be made available to editors and reviewers. We strongly encourage code deposition in a community repository (e.g. GitHub). See the Nature Portfolio guidelines for submitting code & software for further information.

Data

Policy information about availability of data

All manuscripts must include a data availability statement. This statement should provide the following information, where applicable:

- Accession codes, unique identifiers, or web links for publicly available datasets
- A description of any restrictions on data availability
- For clinical datasets or third party data, please ensure that the statement adheres to our policy

The data that support the findings of this study are presented in the paper and Supplementary data.

Research involving human participants, their data, or biological material

Policy information about studies with human participants or human data. See also policy information about sex, gender (identity/presentation), and sexual orientation and race, ethnicity and racism.

Reporting on sex and gender	*Use the terms sex (biological attribute) and gender (shaped by social and cultural circumstances) carefully in order to avoid confusing both terms. Indicate if findings apply to only one sex or gender; describe whether sex and gender were considered in study design; whether sex and/or gender was determined based on self-reporting or assigned and methods used. Provide in the source data disaggregated sex and gender data, where this information has been collected, and if consent has been obtained for sharing of individual-level data; provide overall numbers in this Reporting Summary. Please state if this information has not been collected. Report sex- and gender-based analyses where performed, justify reasons for lack of sex- and gender-based analysis.*
Reporting on race, ethnicity, or other socially relevant groupings	*Please specify the socially constructed or socially relevant categorization variable(s) used in your manuscript and explain why they were used. Please note that such variables should not be used as proxies for other socially constructed/relevant variables (for example, race or ethnicity should not be used as a proxy for socioeconomic status). Provide clear definitions of the relevant terms used, how they were provided (by the participants/respondents, the researchers, or third parties), and the method(s) used to classify people into the different categories (e.g. self-report, census or administrative data, social media data, etc.) Please provide details about how you controlled for confounding variables in your analyses.*
Population characteristics	*Describe the covariate-relevant population characteristics of the human research participants (e.g. age, genotypic information, past and current diagnosis and treatment categories). If you filled out the behavioural & social sciences study design questions and have nothing to add here, write "See above."*
Recruitment	*Describe how participants were recruited. Outline any potential self-selection bias or other biases that may be present and how these are likely to impact results.*
Ethics oversight	*Identify the organization(s) that approved the study protocol.*

Note that full information on the approval of the study protocol must also be provided in the manuscript.

Field-specific reporting

Please select the one below that is the best fit for your research. If you are not sure, read the appropriate sections before making your selection.

☒ Life sciences ☐ Behavioural & social sciences ☐ Ecological, evolutionary & environmental sciences

For a reference copy of the document with all sections, see nature.com/documents/nr-reporting-summary-flat.pdf

Life sciences study design

All studies must disclose on these points even when the disclosure is negative.

Sample size	In vitro experiments: Sample size of at least three was used in most experiment for statistical analysis, for cell viability sample size of four were used<br>For in vivo experiments: Sample size of five were used for statistical analysis.
Data exclusions	No data were excluded.
Replication	We repeated in vitro experiment at least 2- 3 times, and confirmed the reproducibility of the data.
Randomization	No method of randomization was used for in vitro experiments.<br>Method of randomization was used in in vivo experiments where mice were randomized and sorted based on the BLI signal for further treatment purpose.
Blinding	*Describe whether the investigators were blinded to group allocation during data collection and/or analysis. If blinding was not possible, describe why OR explain why blinding was not relevant to your study.*

Reporting for specific materials, systems and methods

We require information from authors about some types of materials, experimental systems and methods used in many studies. Here, indicate whether each material, system or method listed is relevant to your study. If you are not sure if a list item applies to your research, read the appropriate section before selecting a response.

Materials & experimental systems

n/a	Involved in the study	
☐	☒	Antibodies
☐	☒	Eukaryotic cell lines
☒	☐	Palaeontology and archaeology
☐	☒	Animals and other organisms
☒	☐	Clinical data
☒	☐	Dual use research of concern
☒	☐	Plants

Methods

n/a	Involved in the study	
☒	☐	ChIP-seq
☐	☒	Flow cytometry
☒	☐	MRI-based neuroimaging

Antibodies

Antibodies used	Please see the Supplementary Table S1 for the list of antibodies, supplier name and catalog numbers.
Validation	All antibodies are commercially available and have been validated by the companies.<br>Anti-OSMR antibodies used for in vitro and in vivo treatments were screened and validated in our previous publication (PMID: 34380633).

Eukaryotic cell lines

Policy information about cell lines and Sex and Gender in Research

Cell line source(s)	A2780 cell line was purchased from NCI-DCTD tumor repository and A2780 cisplatin-resistant (A2780-CisR) human ovarian cancer cell lines was purchased from European Collection of Authenticated Cell Cultures (ECACC)-Sigma Aldrich. PE01 (BRCA2-deficient, platinum sensitive), and PE04 (BRCA2-proficient, platinum resistant) were kindly provided by Daniela E Matei, Northwestern University, Chicago, Illinois, USA. OVCAR8 cell line was purchased from National Cancer Institute (NCI). OVCAR8 cisplatin-resistant (OVCAR8-CisR) cells were developed from OVCAR8 parental cell line by continuous exposure to increasing dose of cisplatin (Selleckchem, TX, USA) for a period of 12 months. MCW-OV-SL-3 ovarian cancer cell line was established and characterized in our laboratory as described previously (PMID: 35205706 and PMID: 35626175).
Authentication	Each cell line was authenticated by short tandem repeat (STR) profiling (IDEXX BioAnalytics).
Mycoplasma contamination	All cell lines were tested for Mycoplasma using MycoSensor PCR Assay kit (Agilent, Santa Clara, CA).
Commonly misidentified lines<br>(See ICLAC register)	N/A

Animals and other research organisms

Policy information about studies involving animals; ARRIVE guidelines recommended for reporting animal research, and Sex and Gender in Research

Laboratory animals	4-6-week-old athymic female nude mice (Nu/Nu) (Envigo, Madison, WI, USA)
Wild animals	No
Reporting on sex	Female
Field-collected samples	No
Ethics oversight	All animal work was done in accordance with protocol approved by the Institutional Animal Care and Use Committee (IACUC) at the Medical College of Wisconsin (AUA00005439)

Note that full information on the approval of the study protocol must also be provided in the manuscript.

Plants

Seed stocks	N/A
Novel plant genotypes	N/A
Authentication	N/A

Flow Cytometry

Plots

Confirm that:

☒ The axis labels state the marker and fluorochrome used (e.g. CD4-FITC).

☒ The axis scales are clearly visible. Include numbers along axes only for bottom left plot of group (a 'group' is an analysis of identical markers).

☒ All plots are contour plots with outliers or pseudocolor plots.

☒ A numerical value for number of cells or percentage (with statistics) is provided.

Methodology

Sample preparation	Please see Methods section for cell viability sample preparation.
Instrument	BD FACS-Celesta (BD Bioscience)
Software	Collection of data: BD FACSDiva software (BD Bioscience)<br>Data Analysis: FlowJo v10.9.0
Cell population abundance	Cells were not sorted
Gating strategy	FSC/SSC gating is about 80-85% of total population. Gating on positive/negative were determined by unstained control.<br>Usually <10^2 is considered negative and >10^2 is considered as positive

☐ Tick this box to confirm that a figure exemplifying the gating strategy is provided in the Supplementary Information.
